# TNC-targeted CAR-macrophage therapy alleviates liver fibrosis in mice

**DOI:** 10.1186/s40779-025-00667-3

**Published:** 2025-11-11

**Authors:** Kai-Zhao Chen, Zi-Yang Lin, Long-Jun Chen, You-Xi Zhou, Wei Zhang, Hao-Yang Wan, Yong-Kun Huo, Qi Fu, Zi-Qing Gao, Hong-Wei Cheng, Xiao-Dong Ma, Shuai-Shuai Zhang

**Affiliations:** 1 Key Laboratory of Brain, Cognition and Education Sciences, Institute for Brain Research and Rehabilitation, Guangdong Key Laboratory of Mental Health and Cognitive Science, Ministry of Education, South China Normal University, Guangzhou, 510631 China; 2 Zhuhai UM Science and Technology Research Institute, University of Macau, Macau, 999078 China; 3 State Key Laboratory of Vaccine for Infectious Diseases, Xiang an Biomedicine Laboratory, National Innovation Platform for Industry-Education Integration in Vaccine Research, Fujian Engineering Research Center of Molecular Theranostic Technology, Center for Molecular Imaging and Translational Medicine, School of Public Health, Xiamen University, Xiamen, 361102 Fujian China

**Keywords:** Liver fibrosis, Tenascin-C (TNC), Chimeric antigen receptor (CAR), Macrophage, Hepatic stellate cells (HSCs)

## Abstract

**Background:**

Tenascin-C (TNC) is an extracellular matrix (ECM) protein involved in tissue damage and fibrosis. Chimeric antigen receptor (CAR) cell therapy is a novel therapeutic approach that has attracted increasing attention in recent years. Here, we engineered CAR-macrophages targeting TNC (TNC-CAR-Ms) and explored the underlying mechanism through which TNC-CAR-Ms treat liver fibrosis.

**Methods:**

The role of TNC in liver fibrosis was studied in established *Tnc* knockout (KO) and littermate control mice. A TNC-targeted single-chain variable fragment (scFv) was designed to generate TNC-CAR-Ms and evaluate their biological function. The phagocytosis and killing effects of TNC-CAR-Ms were tested in vitro, while the antifibrotic efficacy and safety of TNC-CAR-Ms were evaluated in vivo. The underlying mechanism through which TNC-CAR-Ms treat liver fibrosis was investigated by Western blotting, flow cytometry, and RNA sequencing.

**Results:**

TNC expression was significantly upregulated in the liver and activated hepatic stellate cells (HSCs) in carbon tetrachloride (CCl_4_)-treated mice. Animal studies showed that *Tnc* KO protects mice from CCl_4_-induced liver damage and fibrosis. Upon demonstrating their ability to engulf and kill activated HSCs, we intravenously administered TNC-CAR-Ms to fibrotic mice and found that TNC-CAR-Ms significantly reduced liver fibrosis. Mechanistically, TNC-CAR-Ms specifically migrated to liver tissues, potently reduced TNC expression, and decreased the activity of the Toll-like receptor 4 (TLR4)/nuclear factor kappa-B (NF-κB) and integrin/focal adhesion kinase (FAK) signaling pathway. In addition, TNC-CAR-Ms significantly modified the hepatic immune microenvironment, characterized mainly by an increase in the numbers of M2-polarized macrophages and CD8^+^ T cells in the liver. Finally, in CCl_4_-treated mice, the depletion of CD8^+^ T cells with an anti-CD8α antibody significantly impaired the antifibrotic effect of TNC-CAR-Ms.

**Conclusions:**

Our proof-of-concept study demonstrates the therapeutic potential of TNC-CAR-Ms in alleviating liver fibrosis and may inform the development of future therapeutic strategies for the treatment of a range of liver diseases with a fibrotic phenotype.

**Supplementary Information:**

The online version contains supplementary material available at 10.1186/s40779-025-00667-3.

## Background

Liver fibrosis is an inevitable stage of chronic liver disease and currently has limited treatment options. If not treated on time, all patients with chronic liver disease progress to advanced fibrosis, resulting in decompensated cirrhosis or the development of hepatocellular carcinoma, eventually increasing the risk of liver-related mortality [1]. A common pathological manifestation of hepatic fibrosis is the secretion of extracellular matrix (ECM) by myofibroblasts in the hepatic infiltrate. Excessive deposition of ECM changes the structure of the liver tissue and worsens liver function, and is therefore critical for the development of hepatic fibrosis [2, 3]. Activated hepatic stellate cells (HSCs) are the primary source of myofibroblasts and are a driving force of fibrogenesis, which is often a sign of the progression of hepatic fibrosis [4, 5]. Complex cellular networks play key roles in maintaining homeostasis in the liver, and the balance between the activation and deactivation of HSCs determines the overall status of hepatic fibrosis. Despite the ongoing development of various measures aimed at inhibiting the activity of fibrogenic factors, promoting the degradation of collagen fibers, genetically eliminating liver fibroblasts, and improving hepatic microcirculation, effective clinical interventions targeting HSC activation remain limited in the treatment of liver fibrosis [6, 7].

Tenascin-C (TNC) is a hexameric ECM protein with cell signaling properties. Structurally, TNC consists of an N-terminal tenascin assembly sequence, numerous fibronectin type III repeat sequences, variable epidermal growth factor-like domains, and a fibrinogen-like domain at the C-terminus [8]. As a so-called ECM protein, TNC is not a necessary structural component in the ECM, but it can bind to ECM structural proteins and cell surface receptors such as integrins and Toll-like receptor 4 (TLR4) [9–11]. TNC is generally expressed at low or undetectable levels in normal adult tissues and is strictly restricted to areas essential for cell renewal, plasticity, and tissue remodeling [12]. In contrast, higher TNC expression is observed in various tumors and fibrotic lesions, including systemic sclerosis, lung injury and fibrosis, and kidney fibrosis [13–17]. Although studies have suggested a role for TNC in liver fibrosis [18, 19], the mechanistic link between TNC and liver fibrosis progression, particularly that mediated by activated HSCs, has not been established, and, more importantly, TNC has never been therapeutically targeted using chimeric antigen receptor-macrophages (CAR-Ms).

Recently, TNC-targeted CAR-T cells have been promising immunotherapies for pediatric sarcoma and brain tumors, and eliminate autoreactive B cells from rheumatoid arthritis patients [20, 21]. Despite the significant advancements and widespread application of adoptive cell therapy utilizing CAR technology in malignancies and autoimmune diseases, the application of CAR technology in hepatic fibrosis remains underexplored [22, 23]. Moreover, CAR-T-cell therapy still has several limitations to overcome in autologous and allogeneic settings, such as practicality and toxicity issues; thus, alternative CAR cell therapies are being developed [24]. One alternative is the use of innate immune macrophages, whose chemical depletion or genetic manipulation has been shown to decrease liver fibrosis in the fibrosis resolution stage; thus, these cells hold promise for the development of new therapeutic strategies for liver fibrosis [25, 26]. Macrophages can infiltrate solid tumors and fibrotic tissues better than T cells. T cells kill target cells by triggering apoptosis, but CAR-Ms phagocytose target cells and then secrete cytokines that remodel the surrounding environment. In the liver, macrophages are broadly defined as either resident Kupffer cells or monocyte-derived macrophages [27, 28]. The innate immunity conferred by macrophages, their ability to scavenge necrotic cells, and their ability to modulate the local microenvironment, coupled with their reduced risk of inducing cytokine release syndrome and increased potential for allogeneic utilization compared with CAR-T cells, render CAR-Ms compelling cell therapeutic candidates for the treatment of liver fibrosis [29, 30].

In this study, we aimed to evaluate whether CAR-Ms targeting TNC (TNC-CAR-Ms) can alleviate liver fibrosis and explore the underlying antifibrotic mechanism of TNC-CAR-Ms in vivo. The purpose of our study was to present an innovative approach for alleviating liver fibrosis through the use of genetically engineered macrophages, ultimately providing new insight and evidence for the treatment of liver fibrosis.

## Methods

### Animal experiments

Animal experiments were performed in accordance with international guidelines for laboratory animals. All animal experimental procedures were approved by the Institutional Animal Care and Use Committee of South China Normal University (SCNU-BRR-2024-037). Wild-type (WT) C57BL/6J mice (7–8 weeks old, male, *n* = 170) were housed under specific pathogen-free conditions (temperature: 18–22 °C; humidity: 40–60%; 12-h light/dark cycle). *Tnc* knockout (KO) mice (strain background: C57BL/6J) were generated using CRISPR/Cas9-mediated gene editing technology targeting exons 3–5 of the *Tnc* gene by Cyagen Biotechnology Co., Ltd., in China. *Tnc* KO mice and their methods for identifying them can be obtained by contacting the corresponding author. The mice had free access to standard chow and water. Health conditions were monitored and recorded daily.

Liver fibrosis models were established in WT and *Tnc* KO mice via the intraperitoneal injection of carbon tetrachloride (CCl_4_). CCl_4_ was prepared as a 15% solution in olive oil (MACKLIN, O815211, Shanghai, China). The experimental group received intraperitoneal injections of CCl_4_ twice weekly at a dose of 0.6 ml/kg body weight for 6 weeks, whereas the control group received the same amount of olive oil. The mice were monitored for weight and behavioral changes throughout the experiment. The mice were divided into the 4 groups to evaluate the antifibrotic efficacy of TNC-CAR-Ms in a liver fibrosis model. Mice in the CCl_4_ + PBS, CCl_4_ + Mock-CAR-Ms (macrophages transduced with empty CAR vectors), and CCl_4_ + TNC-CAR-Ms groups (*n* = 6) were intraperitoneally injected with CCl_4_ (0.6 ml/kg; dissolved in olive oil) twice weekly for 6 weeks and separately injected with PBS, Mock-CAR-Ms (2 × 10^6^ cells in PBS), or TNC-CAR-Ms (2 × 10^6^ cells in PBS) during the last 2 weeks. WT C57BL/6J mice were used as the control group and were injected with olive oil and PBS. The livers were divided into different portions for various analyses: 1) a portion was fixed with 4% paraformaldehyde for the histological analysis; 2) another portion was frozen in liquid nitrogen for RNA and protein extraction and analysis; and 3) the remaining liver tissues were mechanically dissociated to isolate immune cells for flow cytometry analysis. For the long-term cirrhosis model, mice were intraperitoneally injected with CCl_4_ twice weekly for 12 weeks and intravenously infused with PBS, Mock-CAR-Ms, or TNC-CAR-Ms during the last 2 weeks.

For the methionine choline deficient (MCD)-induced nonalcoholic steatohepatitis (NASH) fibrosis model, mice were fed an MCD diet (MD12052; Jiangsu Medicine, China) for 4 weeks and then were infused with PBS, Mock-CAR-Ms, or TNC-CAR-Ms for 2 weeks. Mice fed a normal diet were used as the control group.

Mice were intraperitoneally injected with CCl_4_ twice weekly for 6 weeks and intravenously administered PBS, Mock-CAR-Ms, TNC-CAR-Ms, or M2-polarized TNC-CAR-Ms for 2 weeks to evaluate the antifibrotic effect of M2-polarized TNC-CAR-Ms in vivo (*n* = 6). TNC-CAR-Ms were treated with interleukin-4 (IL-4; 20 ng/ml, Novoprotein, Suzhou, China) for 24 h to obtain M2-polarized CAR-Ms. The M2 polarization of CAR-Ms was verified by real-time quantitative polymerase chain reaction (RT-qPCR) and flow cytometry.

The mice were intraperitoneally injected with CCl_4_ twice weekly for 6 weeks, and intraperitoneally administered an anti-CD8α monoclonal antibody (Selleck, A2102, Houston, USA) or an isotype control IgG antibody at a dose of 1 mg/kg every 3 d starting 1 d before PBS or TNC-CAR-Ms cell infusion for 2 weeks to determine the involvement of CD8^+^ T cells in the antifibrotic effect of TNC-CAR-Ms in vivo (*n* = 6). The depletion efficiency was confirmed via a flow cytometry analysis of the proportion of CD8^+^ T cells in the spleen. At the endpoint, the mice were sacrificed, and liver tissues were collected for histological assessment.

More details on the methods involved in the research can be found in Additional file 1: Methods.

### Generation of CAR-Ms

The single-chain variable fragment (scFv) sequence of TNC was derived from the small immunoprotein P12 antibody specific for the D domain of TNC (US7968685B2). Afterward, the lentiviral vector carrying the TNC-CAR construct was engineered via restriction enzyme digestion-based molecular cloning, replacing the scFv region of the programmed cell death-ligand 1 (PD-L1)-CAR lentiviral vector encoding an enhanced green fluorescent protein (EGFP)-thosea asigna virus 2A (T2A)-PD-L1 scFv-CD8-4-1BB-CD3ζ expression cassette with a TNC scFv. TNC-CAR-luciferase constructs were generated using In-Fusion cloning (Takara Bio, Japan) to replace the EGFP sequence with the luciferase sequence, which was amplified by PCR from the pLX302 luciferase-V5 puro (47,553, Addgene, USA) vector. In addition, we connected the resistance gene of puromycin via P2A at the C-terminus of the above sequence, which eventually formed the TNC-CAR-EGFP-puro or TNC-CAR-luciferase-puro plasmid, to facilitate enrichment of transfected CAR-Ms cells. A lentiviral vector encoding the CAR sequence was cotransfected with packaging plasmids into HEK293T cells to produce lentiviral particles that would subsequently be used to generate CAR-Ms. Viral supernatants were collected and concentrated after 48 h. Murine macrophages were then transduced with the concentrated lentivirus in the presence of polybrene. After 48–72 h, CAR-expressing macrophages were selected using puromycin resistance. The expression of TNC scFv in CAR-Ms was confirmed by RT-qPCR and flow cytometry.

### In vitro cytotoxicity assay

The cytotoxic potential of CAR-Ms was evaluated using a luciferase-based bioluminescence (BLI) assay in a 96-well white-walled plate, with HSCs and GL261 cells (Cell Resource Center, Peking Union Medical College, China) serving as target cells. HSCs and GL261 cells were genetically modified to express luciferase and seeded at a density of 8000 cells per well in 100 μl of DMEM supplemented with 10% FBS and 1% penicillin/streptomycin. The plate was incubated overnight at 37 °C in a 5% CO_2_ atmosphere to allow for cell attachment. After 12 h, the CAR-Ms were harvested, resuspended in fresh culture medium, and added to the wells at different effector-to-target (E/T) ratios (20:1, 10:1, 5:1, and 1:1) in 100 μl of medium, resulting in a total volume per well of 200 μl. Luciferase-based cytotoxicity was measured at 12, 24, and 36 h after coculture initiation. At each time point, D-luciferin was added to each well at a final concentration of 1.5 mg/ml in 100 μl of PBS, and the plate was incubated at 37 °C for 10 min to allow substrate uptake. A Luciferase Reporter Gene Assay Kit (11401ES80, Yeasen, China) was used to measure the killing efficiency, and the percentage of cell lysis was calculated using the following formula: % specific lysis = [(control BLI − sample BLI)/(control BLI − background BLI)] × 100%, where control BLI refers to the baseline BLI of target cells alone, sample BLI corresponds to the BLI of target cells cocultured with CAR-Ms, and background BLI represents the BLI from wells without cells. All experiments were conducted in 3 independent replicates to ensure the reliability and reproducibility of the statistics, and a statistical analysis was subsequently conducted using GraphPad Prism software.

### Fluorescence-based phagocytosis assay

A total of 1 × 10^5^ activated HSCs (EGFP^+^) were incubated in serum-free medium for 12 h, after which 5 × 10^5^ Mock-CAR-Ms or TNC-CAR-Ms (5:1 E/T ratio) were added and incubated for 3 h to observe the phagocytosis process. Changes in the fluorescence of activated HSCs and EGFP-loaded macrophages were monitored, and coculture images were captured with a microscope.

### Fluorescence-activated cell sorting-based phagocytosis assay

A total of 2 × 10^5^ Mock-CAR-Ms or TNC-CAR-Ms were plated in each well of a 12-well culture plate to study phagocytosis. The next day, 1 × 10^5^ EGFP-labeled activated HSCs (2:1 E/T ratio) and 10% FBS were added to the media and incubated for 3 h. After coculture, the Mock-CAR-Ms or TNC-CAR-Ms were harvested and stained with anti-F4/80 phycoerythrin (PE, BioLegend, USA), and phagocytosis was then determined by flow cytometry analysis. Phagocytic activity is denoted as the percentage of EGFP^+^ events within the F4/80^+^ population.

### Flow cytometry analysis

Flow cytometry can be used to accurately assess cell surface markers and cytokine production using preconjugated fluorophore-labeled antibodies. Briefly, cells were collected, washed twice with PBS, and resuspended at an appropriate concentration. Preconjugated antibodies were then added to the tubes containing the washed cells, and the cells were incubated in the dark for 30 min at 24 °C. After being incubated overnight, the cells were washed twice with PBS and resuspended in flow cytometry buffer for detection. TNC-CAR expression in bone marrow-derived macrophages (BMDMs) was detected with Biotin-Protein L (GenScript) and a fluorescein isothiocyanate (FITC)-labeled secondary antibody. Monocytes and macrophages were detected using the following antibodies: anti-CD11b FITC (Invitrogen, USA), anti-F4/80 PE (BD Biosciences, USA), anti-CD80 allophycocyanin (APC, Invitrogen, USA), and anti-CD206 APC (BioLegend, USA). T lymphocytes were detected with the following antibodies: anti-CD3 PE (Invitrogen, USA), anti-CD4 FITC (BioLegend, USA), and anti-CD8a APC (BioLegend, USA) (Additional file 1: Table S1). The samples were analyzed using a Beckman flow cytometer, and the data were processed with FlowJo software.

### Ex vivo BLI imaging

BLI imaging was performed using the Clinx-IVScope 8000 series imaging system to monitor the distribution of luciferase-expressing TNC-CAR-Ms in mice. Three days after the TNC-CAR-Ms infusion, the mice were intraperitoneally injected with the D-luciferin potassium salt (Meilun Star, MB1834) at a dose of 150 mg/kg body weight and allowed to rest for 10 min to ensure optimal substrate distribution. Following luciferin administration, the mice were anesthetized using isoflurane and euthanized. Mouse organs, including heart, brain, spleen, liver, lung, and kidney, were collected and positioned in a 10 cm dish to capture fluorescence images in the imaging chamber of the Clinx-IVScope 8000 system. Imaging parameters, including the exposure time, binning, and field of view, were optimized to achieve a high signal-to-noise ratio. Bioluminescent signals were detected and quantified using the system’s dedicated analysis software. The data are presented as total photon flux (photons per second) within defined regions of interest. All the imaging sessions were conducted under standardized conditions to ensure reproducibility.

### RNA-seq analysis

Total RNA extracted from tissues was purified using a commercial kit, and sample quality was verified through both a spectrophotometric analysis (NanoDrop) and electrophoretic assessment (Agilent 2100 Bioanalyzer). Only RNA samples with integrity scores exceeding 7.0 were advanced to the next step. Strand-specific libraries were constructed from polyadenylated transcripts using standard protocols. After quantification, the libraries were multiplexed and sequenced on the Illumina platform to yield 150 bp paired-end reads. Quality control of the raw data was performed using FastQC, followed by adaptor and low-quality base removal via Trimmomatic. Reads passing the quality filters were aligned to the mouse reference genome using the STAR aligner. Gene expression was quantified with featureCounts. Differential expression was assessed using the DESeq2 package, with an adjusted *P*-value (false discovery rate, FDR) threshold of 0.05 for significance. Downstream analyses included pathway and Gene Ontology analyses to interpret the functions of the differentially expressed transcripts.

### Statistical analysis

The data are shown as the means ± standard deviations. For statistical analyses of the results, at least 3 biological replicate samples were included. Statistical analyses were conducted with GraphPad Prism 8.0 software. Comparisons between two groups were conducted using the unpaired two-tailed Student’s *t*-test. For comparisons of more than two groups, one-way analysis of variance (ANOVA) with Tukey’s post hoc test was applied to analyze the differences. A *P-*value < 0.05 was considered to indicate statistical significance. Statistical significance is indicated as follows: ^*^P < 0.05, ^**^P < 0.01, ^***^P < 0.001.

## Results

### TNC deficiency protects mice from CCl_4_-induced hepatic fibrosis

We first investigated *TNC* expression in clinical patients with different liver fibrosis stages. The results showed that *TNC* expression was increased in most fibrotic tissues compared with normal control tissues (Additional file 1: Fig. S1a). *TNC* expression was significantly higher in patients with advanced fibrosis than in patients with early fibrosis (Additional file 1: Fig. S1a, b). Our immunohistochemical analysis of liver tissue samples from cirrhosis patients and fibrosis-free patients also confirmed the increased expression of the TNC protein in fibrosis patients (Additional file 1: Fig. S1c). The survival analysis revealed that high TNC expression is associated with shorter overall survival of patients with cirrhosis (Additional file 1: Fig. S1d). The correlation analysis further revealed a strong positive correlation between *TNC* expression and transforming growth factor-β (*TGFβ2*) expression (*R* > 0.30, *P* < 0.001), indicating the potential fibrogenic function of TNC in fibrosis progression (Additional file 1: Fig. S1e).

We next investigated the expression of TNC in mouse models of hepatic fibrosis. CCl_4_ is a potent hepatotoxin that has been extensively used for chemical-induced hepatic fibrosis in mice. In the public Gene Expression Omnibus database, we first found that the expression of the *Tnc* gene was significantly increased in the liver tissue of mice with CCl_4_-induced fibrosis (Additional file 1: Fig. S2a). In addition, no significant changes in the expression of the genes encoding tenascin-W (*Tnn*), tenascin-R (*Tnr*), and tenascin-X (*Tnxb*), which are other members of the tenascin family, were observed (Additional file 1: Fig. S2a). Previous evidence has indicated that several integrins, including integrins αvβ3, αvβ6, α2β1, α7β1, α8β1, and α9β1, are likely TNC receptors [31]. Through a bioinformatics analysis, we also observed that the expression of most genes encoding TNC receptors, such as *Itgav*, *Itga2*, *Itga8*, *Itga9*, *Itgb1*, and *Itgb6*, but not *Itga7* and *Itgb3*, was upregulated in the liver tissues of mice with fibrosis (Additional file 1: Fig. S2b).

**Fig. 1:**
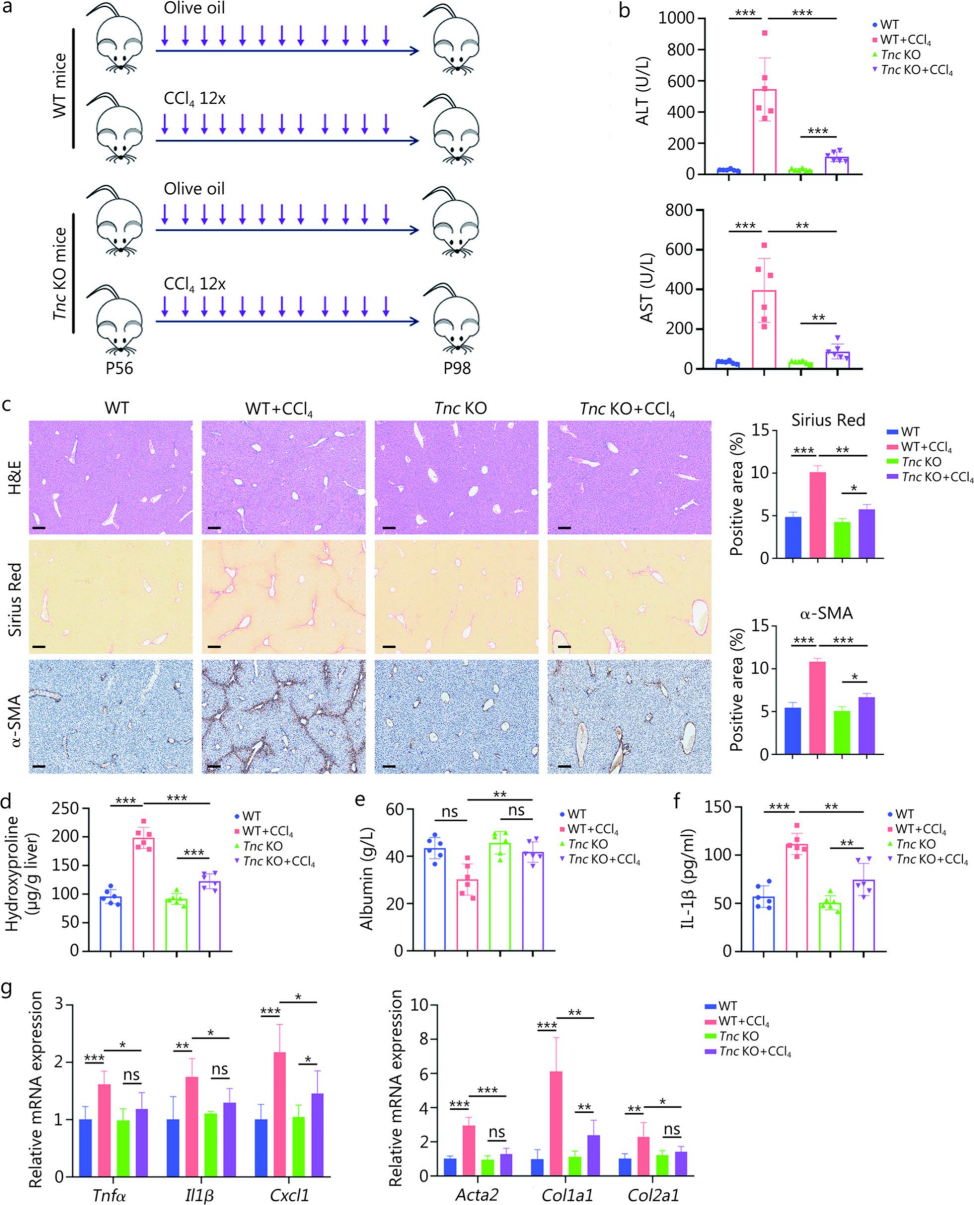
TNC deficiency protects mice from CCl_4_-induced hepatic fibrosis. **a** Schematic of the CCl_4_-induced liver fibrosis models. Wild-type (WT) and *Tnc* gene knockout (*Tnc* KO) mice were intraperitoneally injected with 0.6 ml/kg CCl_4_ twice weekly for 6 weeks (*n* = 6). Olive oil was used as a control treatment. The 12 × refers to the repeated injections of CCl_4_ for 12 times. P56 and P98 indicate the mice’s age in postnatal days (day 56 and day 98, respectively).** b** Serum ALT and AST levels. After the final CCl_4_ injection, blood samples were collected, and serum was obtained by centrifugation. **c** Liver tissues from WT, WT + CCl_4_, *Tnc* KO, and *Tnc* KO + CCl_4_ mice were sectioned and subjected to H&E staining, Sirius Red staining, and α-SMA IHC staining. Quantification of Sirius Red staining and α-SMA IHC staining was performed using ImageJ software. Scale bar = 200 μm. **d** Measurement of the hydroxyproline content in liver tissues from mice in the WT, WT + CCl_4_, *Tnc* KO, and *Tnc* KO + CCl_4_ groups. **e** Measurement of serum albumin levels in WT, WT + CCl_4_, *Tnc* KO and *Tnc* KO + CCl_4_ mice. **f** ELISA of IL-1β protein levels in the serum of mice from the WT, WT + CCl_4_, *Tnc* KO, and *Tnc* KO + CCl_4_ groups (*n* = 6). **g** RT-qPCR analysis of the mRNA expression levels of proinflammatory genes (*Tnfα*, *Il1β,* and *Cxcl1*) and profibrogenic genes (*Acta2*, *Col1a1,* and *Col2a1*) in liver tissues from WT, WT + CCl_4_, *Tnc* KO, and *Tnc* KO + CCl_4_ mice. Data are presented as mean ± SD. ^*^P < 0.05, ^**^P < 0.01, ^***^P < 0.001, ns non-significant. ALT alanine aminotransferase, AST aspartate aminotransferase, H&E hematoxylin and eosin, α-SMA α-smooth muscle actin, IL-1β interleukin-1β, Tnfα tumor necrosis factor-α, Cxcl1 chemokine (C-X-C motif) ligand 1, Acta2 actin alpha 2, Col1a1 collagen type I alpha 1 chain, Col2a1 collagen type II alpha 1 chain, IHC immunohistochemistry, ELISA enzyme-linked immunosorbent assay, RT-qPCR reverse transcription quantitative polymerase chain reaction, TNC tenascin-C

Hepatic fibrosis was then induced in *Tnc* KO mice and their WT littermates using CCl_4_ to investigate the potential role of TNC in hepatic fibrosis (Fig. 1a). All groups of mice had similar body weights at the end of the experiments (Additional file 1: Fig. S2c). However, compared with WT mice, *Tnc* KO mice showed a significant reduction in hepatic injury, as indicated by assessments of serum alanine aminotransferase (ALT) levels, aspartate aminotransferase (AST) levels, liver morphology, and hematoxylin and eosin (H&E) staining at 6 weeks after the CCl_4_ injection (Fig. 1b, c; Additional file 1: S2d). More importantly, Sirius Red staining, α-smooth muscle actin (α-SMA) IHC, and hydroxyproline detection revealed reduced collagen deposition in the livers of *Tnc* KO mice (Fig. 1c, d). Serum albumin levels in the *Tnc* KO mice were notably increased at 6 weeks after the CCl_4_ injection, indicating a reduction in liver damage (Fig. 1e). Enzyme-linked immunosorbent assay detection of serum IL1β levels also revealed a reduction in inflammation in *Tnc* KO mice at 6 weeks after the CCl_4_ injection (Fig. 1f). These observations were supported by the RT-qPCR analysis that showed the downregulated expression of proinflammatory factors (*Tnfα, Il1β*, and *Cxcl1*) and fibrogenic genes (*Acta2*, *Col1a1*, and *Col2a1*) in the livers of *Tnc* KO mice (Fig. 1g). Following the 6-week CCl_4_ injections, TNC protein expression levels in the livers of WT mice increased, and TLR4, p-NF-κB, integrin αV, and p-FAK protein expression levels significantly increased, indicating the upregulated activity of the TLR4/nuclear factor kappa-B (NF-κB) and integrin/focal adhesion kinase (FAK) pathways, 2 classical signaling pathways involved in inflammation and fibrosis (Additional file 1: Fig. S2e). Intriguingly, compared with WT mice, *Tnc* KO mice exhibited a substantial loss of TNC expression, in parallel with the decreased expression levels of TLR4, p-NF-κB, integrin αV, and p-FAK protein, indicating the downregulated activity of the TLR4/NF-κB and integrin/FAK pathways (Additional file 1: Fig. S2e). Overall, these findings suggest that TNC may contribute to hepatic fibrosis and serve as a potential therapeutic target.

### Generation of TNC-CAR-Ms and evaluation of antigen-dependent phagocytosis

**Fig. 2:**
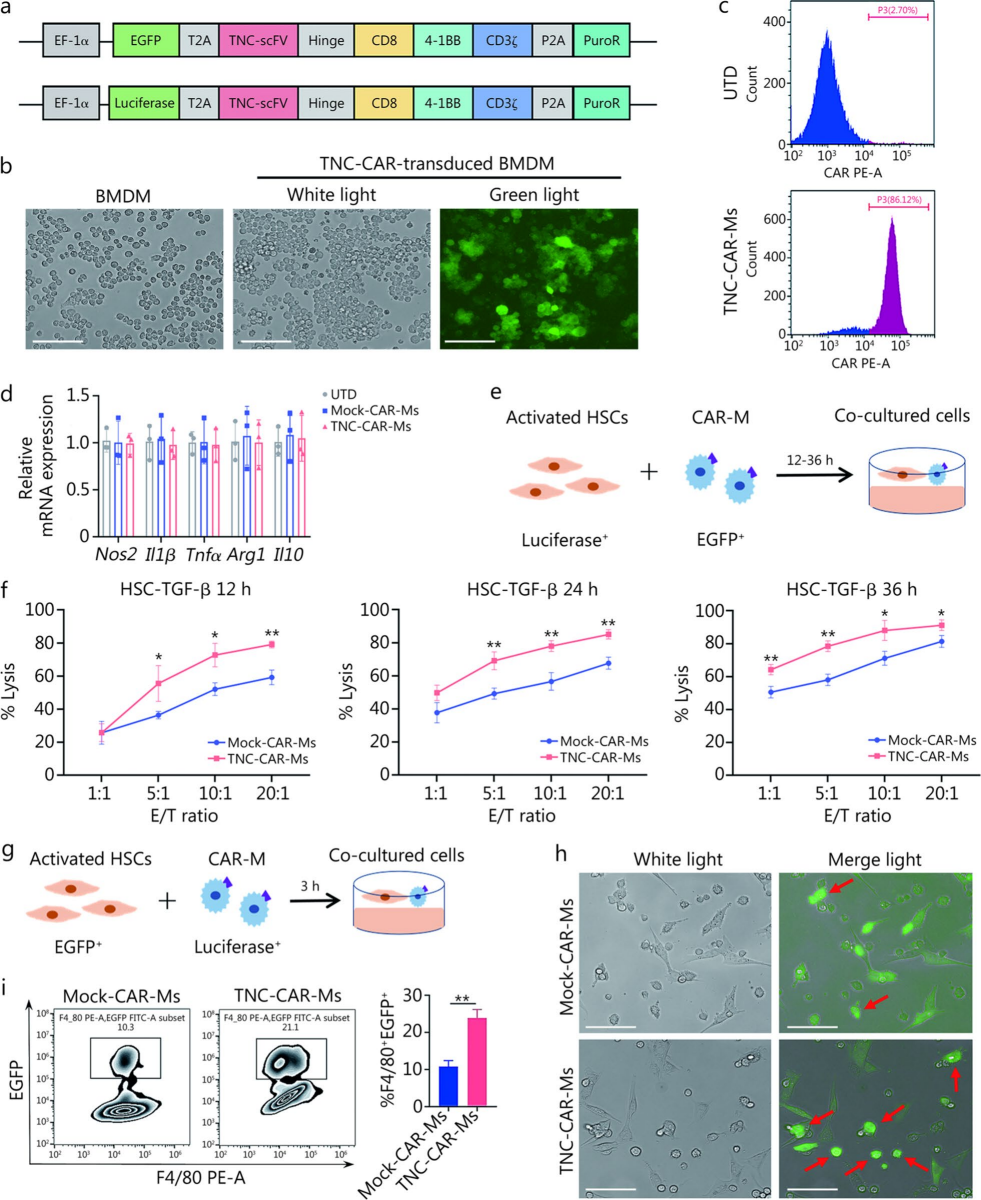
Generation and functional validation of CAR-Ms targeting TNC in vitro. **a** Schematic diagram of the TNC-CAR vector sequence. **b** Microscopy images of TNC-CAR-transfected BMDMs and non-transfected cells, where the TNC-CAR-transfected cells exhibited green fluorescence under a fluorescence microscope because they expressed the EGFP. Scale bar = 100 μm. **c** TNC scFv expression in TNC-CAR-Ms and non-transfected (UTD) control cells was detected using flow cytometry. TNC-CAR-transfected cells were incubated with biotinylated protein L antibodies, followed by staining with fluorescent dye-labeled secondary antibodies. **d** RT-qPCR analysis of the expression levels of macrophage polarization-related genes, including the M1 markers *Nos2*, *Il1β*, *Tnfα*, and the M2 marker *Arg1*, *Il10*, in Mock-CAR-Ms, TNC-CAR-Ms, and UTD macrophages. **e** Illustration of the HSC and CAR-Ms coculture system for the killing assay. **f** In vitro killing assay. TGF-β-stimulated HSCs (stably expressing luciferase) were cocultured with Mock-CAR-Ms and TNC-CAR-Ms. After coculture for 12, 24, and 36 h, cell viability and the fluorescence intensity were assessed to evaluate the cytotoxicity of TNC-CAR-Ms and Mock-CAR-Ms toward HSCs. ^*^P < 0.05*,* ^**^P < 0.01, vs. Mock-CAR-Ms. **g** Illustration of the HSC and CAR-Ms coculture system for the phagocytosis assay. **h** Fluorescence microscopy images of CAR-Ms and HSCs. The CAR-Ms appeared round and nonfluorescent before they were cocultured with EGFP-labeled fusiform HSCs. The merged signal refers to the combination of white light and green fluorescence (EGFP-labeled HSCs). Scale bar = 100 µm. **i** Detection of phagocytic activity (F4/80^+^EGFP^+^ cells) in the coculture system using flow cytometry. Data are presented as mean ± SD. ^**^P < 0.01. EGFP enhanced green fluorescent protein, T2A thosea asigna virus 2A peptide, scFv single-chain fragment variable, CD8 cluster of differentiation 8, CD3 cluster of differentiation 3, P2A porcine teschovirus-1 2A peptide, PuroR puromycin resistance gene, BMDMs bone marrow-derived macrophages, PE phycoerythrin, Nos2 nitric oxide synthase 2, Il1β interleukin-1β, Tnfα tumor necrosis factor-α, Arg1 arginase 1, Il10 interleukin-10, HSCs hepatic stellate cells, TGF-β transforming growth factor-β, RT-qPCR reverse transcription quantitative polymerase chain reaction, TNC tenascin-C, CAR-Ms chimeric antigen receptor-macrophages

We next designed a second-generation CAR structure consisting mainly of an anti-TNC scFv linked to the CD8α transmembrane region, the intracellular costimulatory domain 4-1BB (CD137), and the CD3ζ signaling domain in a lentiviral vector harboring an *EGFP* or *luciferase* gene, abbreviated as TNC-CAR (Fig. 2a). CD3ζ signaling domains are canonical signaling molecules for antibody-dependent cellular phagocytosis in macrophages [32]. We transduced mouse RAW264.7 macrophages and immortalized BMDMs with the abovementioned anti-TNC CAR. After lentivirus infection, TNC-CAR-engineered macrophages were visualized by immunofluorescence (IF) staining and microscopy for EGFP signals (Fig. 2b; Additional file 1: Fig. S3a). The expression of the TNC-targeted scFv sequence in BMDMs was verified by flow cytometry (Fig. 2c). These results revealed the successful generation of TNC-CAR-Ms. We performed RT-qPCR to analyze the expression of genes related to M1/M2 polarization in different BMDMs and to investigate the possible functional correlation between lentivirus infection and macrophage polarization (Fig. 2d). The RT-qPCR results showed that neither TNC-CAR transfection nor Mock-CAR transfection changed the polarization characteristics of macrophages compared with those of untransfected cells (Fig. 2d).

Next, we examined whether the killing of target cells by CAR-Ms required the TNC antigen. Single-cell RNA sequencing revealed that *Tnc* was specifically upregulated in HSCs in response to CCl_4_-induced liver fibrosis (Additional file 1: Fig. S3b). Therefore, Mock-CAR-Ms and TNC-CAR-Ms were coincubated with luciferase^+^ murine HSCs in vitro (Fig. 2e). The initial results showed no significant difference between the effects of Mock-CAR-Ms and TNC-CAR-Ms on the death of unstimulated HSCs (Additional file 1: Fig. S3c). Intriguingly, compared with Mock-CAR-Ms, TNC-CAR-Ms killed most TGF-β-stimulated HSCs at different E/T ratios and times (Fig. 2f). TNC antigen induction in TGF-β-treated HSCs was verified by Western blotting (Additional file 1: Fig. S3d). A strong killing effect of TNC-CAR-Ms on TNC antigen-positive target cells was also observed (Additional file 1: Fig. S3e). Additional imaging analysis revealed that the EGFP-labeled HSCs emitted bright green fluorescence in the TNC-CAR-Ms, suggesting that the engulfed HSCs were degraded in the phagolysosome lumen of the TNC-CAR-Ms (Fig. 2g, h). The flow cytometry data indicated a more vital interaction between TNC-CAR-Ms and EGFP-labeled HSCs, as the proportion of F4/80^+^EGFP^+^ cells was 21.1% compared with the proportion of 10.3% for Mock-CAR-Ms and EGFP-labeled HSCs (Fig. 2i). These data suggest that TNC-CAR-Ms can phagocytose activated HSCs and TNC-positive cells in vitro.

### TNC-CAR-Ms alleviate liver fibrosis in multiple experimental animal models

**Fig. 3:**
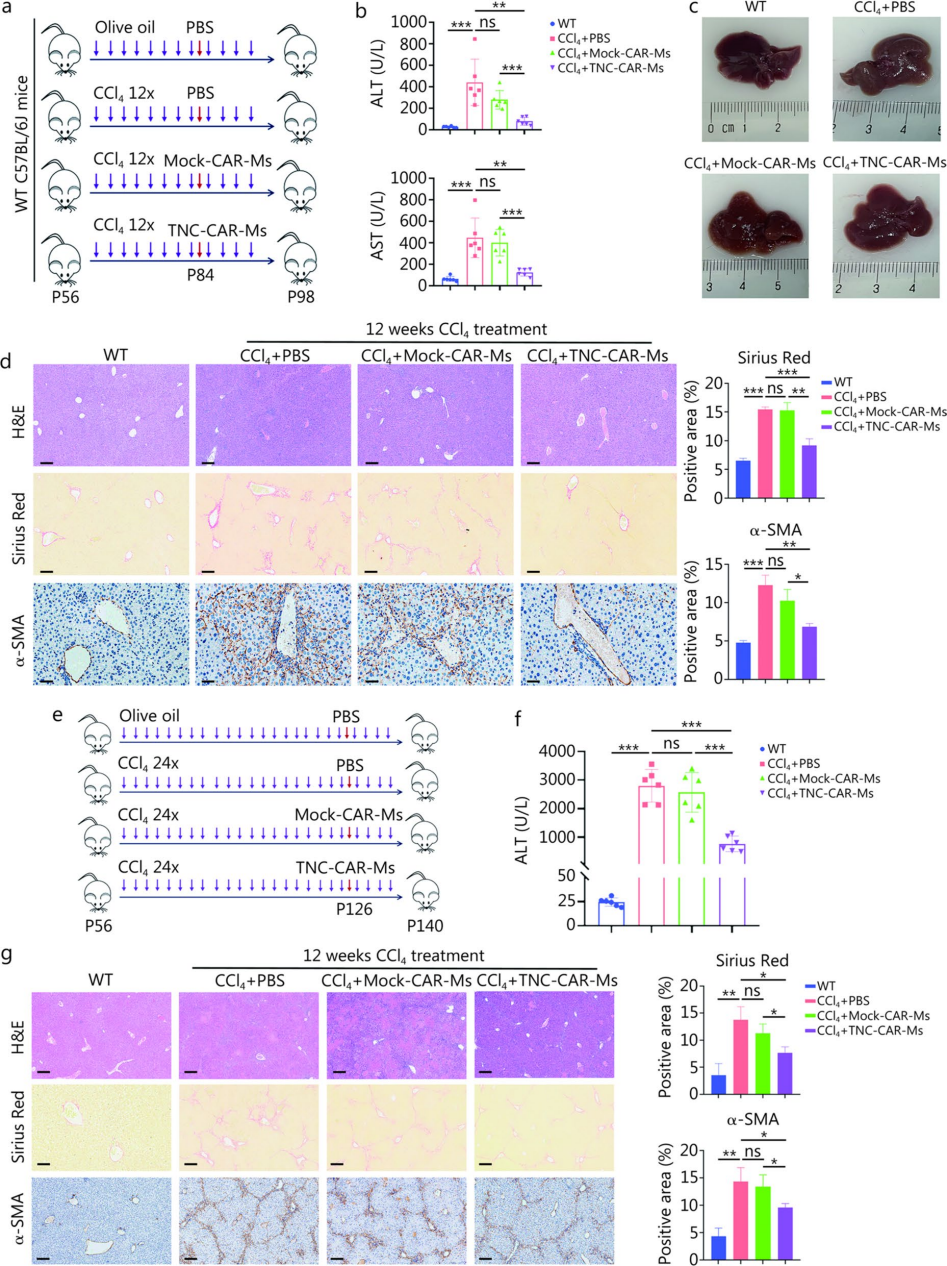
TNC-CAR-Ms exerted antifibrotic effects in vivo. **a** Experimental diagram of CAR-Ms therapy in liver fibrosis mouse models. The CCl_4_ + PBS, CCl_4_ + Mock-CAR-Ms, and CCl_4_ + TNC-CAR-Ms groups were treated with PBS solution, Mock-CAR-Ms, or TNC-CAR-Ms (i.v., 2 × 10^6^ cells/mouse), respectively, 24 h after the 8th CCl_4_ injection. The control group of WT mice was injected with olive oil (*n* = 6). The 12 × refers to the repeated injections of CCl4 for 12 times. P56, P84, and P98 indicate the mice’s age in postnatal days (day 56, day 84, and day 98, respectively). **b** Serum ALT and AST levels in mice from the WT control, CCl_4_ + PBS, CCl_4_ + Mock-CAR-Ms, and CCl_4_ + TNC-CAR-Ms groups. **c** Representative images of liver tissues from mice in the WT control, CCl_4_ + PBS, CCl_4_ + Mock-CAR-Ms, and CCl_4_ + TNC-CAR-Ms groups. **d** Liver tissues were embedded, sectioned, and subjected to H&E staining, Sirius Red staining, and α-SMA IHC staining. Quantification of Sirius Red staining and α-SMA IHC staining was performed using ImageJ software. Scale bar = 200 μm (H&E staining and Sirius Red staining) and 50 μm (α-SMA IHC staining). **e** Experimental diagram of CAR-Ms therapy in cirrhosis models. The CCl_4_ + PBS, CCl_4_ + Mock-CAR-Ms, and CCl_4_ + TNC-CAR-Ms groups were treated with PBS solution, Mock-CAR-Ms, or TNC-CAR-Ms (i.v., 2 × 10^6^ cells/mouse), respectively, 24 h after the 20th CCl_4_ injection. The control group of WT mice was injected with an equivalent dose of olive oil (*n* = 6). The 24 × refers to the repeated injections of CCl_4_ for 24 times. P56, P126, and P140 indicate the mice’s age in postnatal days (day 56, day 126, and day 140, respectively). **f** Serum ALT levels in the WT control, CCl_4_ + PBS, CCl_4_ + Mock-CAR-Ms, and CCl_4_ + TNC-CAR-Ms groups. **g** Liver tissues were embedded, sectioned, and subjected to H&E staining, Sirius Red staining, and α-SMA IHC staining. Quantification of Sirius Red staining and α-SMA IHC staining was performed using ImageJ software. Scale bar = 200 μm. Data are presented as mean ± SD. ^*^P < 0.05, ^**^P < 0.01, ^***^P < 0.001, ns non-significant. ALT alanine aminotransferase, AST aspartate aminotransferase, H&E hematoxylin and eosin, α-SMA α-smooth muscle actin, IHC immunohistochemistry, TNC tenascin-C, CAR-Ms chimeric antigen receptor-macrophages

We evaluated whether TNC-CAR-Ms could reduce liver fibrosis in vivo by first adoptively transferring these cells to WT C57BL/6J mice intravenously for 2 weeks after 4 weeks of CCl_4_ injections (CCl_4_ + TNC-CAR-Ms group; Fig. 3a). The control groups included mice that received PBS after the CCl_4_ injection (the CCl_4_ + PBS group) and mice that received Mock-CAR-Ms after the CCl_4_ injection (the CCl_4_ + Mock-CAR-Ms group). Mice from the CCl_4_ + TNC-CAR-Ms group showed significantly decreased ALT and AST levels, along with significantly increased albumin contents after TNC-CAR-Ms therapy, indicating improved liver function, but mice from the CCl_4_ + Mock-CAR-Ms group showed no significant differences compared with mice from the CCl_4_ + PBS group (Fig. 3b; Additional file 1: Fig. S4a). Further analysis of liver morphology, the hydroxyproline content, and Sirius Red staining, and α-SMA IHC staining showed that Mock-CAR-Ms could not inhibit hepatic fibrosis, whereas the antifibrotic effect of TNC-CAR-Ms therapy was remarkable, with nearly complete remission observed in CCl_4_-induced mice (Fig. 3c, d; Additional file 1: Fig. S4b). We also evaluated the toxicity of TNC-CAR-Ms treatment. After 2 weeks of treatment, TNC-CAR-Ms therapy did not significantly influence the histological morphology of non-liver organs, including the heart, lungs, spleen, and kidneys (Additional file 1: Fig. S4c). Furthermore, mice treated with TNC-CAR-Ms remained active and did not exhibit significant changes in body weight after the CCl_4_ injection (Additional file 1: Fig. S4d). Cytokine release syndrome did not occur in the mice at the time of the assessment (Additional file 1: Fig. S4e).

We determined whether TNC-CAR-Ms attenuate hepatic fibrosis in the later stage of fibrogenesis. The mice were injected twice weekly with CCl_4_ for 12 weeks to simulate hepatic cirrhosis and subsequently infused with TNC-CAR-Ms for another 2 weeks (Fig. 3e). Compared with the other treatments, TNC-CAR-Ms treatment significantly alleviated liver injury and fibrosis, as indicated by serum ALT levels, H&E staining, Sirius Red staining, and α-SMA IHC staining (Fig. 3f, g). The analysis of the survival rate of mice with cirrhosis showed that TNC-CAR-Ms treatment significantly increased the survival time (Additional file 1: Fig. S4f). These results suggest that TNC-CAR-Ms may be a potential therapeutic strategy for cirrhosis.

We also employed a MCD diet-fed mouse model, another common NASH fibrosis model characterized by rapid fibrosis development, to determine the pivotal role of TNC-targeted CAR-Ms in fibrosis resolution (Additional file 1: Fig. S4g). Compared with that in the PBS group, liver damage in the TNC-CAR-Ms group was decreased, as indicated by the serum ALT and AST levels (Additional file 1: Fig. S4h). In the MCD diet-fed mice, body weight was significantly decreased, consistent with the findings of other reports (Additional file 1: Fig. S4i). Notably, compared with PBS, TNC-CAR-Ms, but not Mock-CAR-Ms, improved mouse body weight (Additional file 1: Fig. S4j). In addition, liver injury and fibrosis were clearly decreased in the TNC-CAR-Ms group compared with those in the PBS group, as measured by H&E staining, Sirius Red staining, and α-SMA IHC staining (Additional file 1: Fig. S4k). These results support the positive role of TNC-CAR-Ms in resolving fibrosis.

### TNC-CAR-Ms mainly migrate to the liver and reduce TNC expression in activated HSCs

**Fig. 4:**
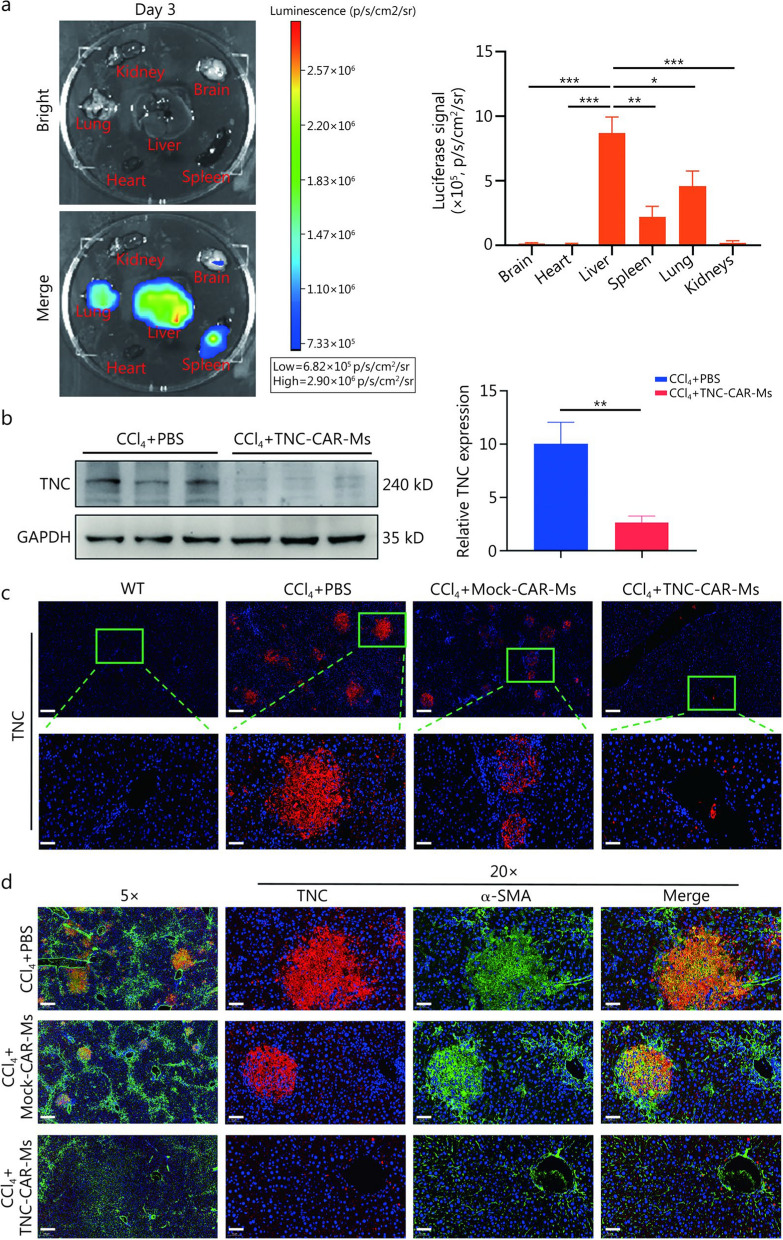
TNC-CAR-Ms migrate to the liver and reduce TNC expression. **a** Assessment of the biological distribution and transport of TNC-CAR-Ms in vivo. Three days after the TNC-CAR-Ms infusion, the mice were anesthetized, euthanized, and tissues were dissected. The distribution of luciferase-expressing TNC-CAR-Ms in brain, heart, liver, spleen, lung, and kidney tissues was monitored with a Clinx-IVScope 8000 imaging system. The distribution of TNC-CAR-Ms in the major organs of mice was quantitatively and statistically analyzed. **b** TNC protein expression in liver tissues from CCl_4_ + PBS and CCl_4_ + TNC-CAR-Ms mice was detected by Western blotting and quantitatively analyzed using Quantity One software. **c** Images of immunofluorescence (IF) staining for the TNC protein in liver tissues from WT, CCl_4_ + PBS, CCl_4_ + Mock-CAR-Ms, and CCl_4_ + TNC-CAR-Ms mice. Scale bar = 200 μm (upper) and 50 μm (lower). **d** Images of IF staining for the TNC and α-SMA proteins in liver tissues from CCl_4_ + PBS, CCl_4_ + Mock-CAR-Ms, and CCl_4_ + TNC-CAR-Ms mice. DAPI was used for nuclear staining. Scale bar = 200 μm (5 ×) and 50 μm (20 ×). Data are presented as mean ± SD. ^*^P < 0.05, ^**^P < 0.01, ^***^P < 0.001. GAPDH glyceraldehyde-3-phosphate dehydrogenase, α-SMA α-smooth muscle actin, DAPI 4',6-diamidino-2-phenylindole, TNC tenascin-C, CAR-Ms chimeric antigen receptor-macrophages

We subsequently assessed the biodistribution and transport of TNC-CAR-Ms after intravenous injection in mice from the CCl_4_ + TNC-CAR-Ms group. Molecular imaging showed that at 3 d after adoptive cell transfer, TNC-CAR-Ms were localized mainly in the liver (Fig. 4a). At 14 d after the infusion, ex vivo imaging revealed the distribution and liver accumulation of CAR-Ms in the mice (Additional file 1: Fig. S5a). However, at 28 d after infusion, no obvious fluorescence was detected in the mice (Additional file 1: Fig. S5a). Consistent with previous studies, we observed the highest fluorescence intensity in the liver after the CAR-Ms injection [29, 32]. In comparison, very faint or no obvious signals were detected in the brain, heart, or kidneys, whereas a modest fluorescence intensity was observed in the lung and spleen (Fig. 4a). Therefore, TNC-CAR-Ms accumulated in the liver tissue within 3 d after adoptive cell transfer.

We next detected and quantified the expression levels of the TNC protein in liver tissues from mice in the CCl_4_ + PBS and CCl_4_ + TNC-CAR-Ms groups. Compared with those in the CCl_4_ + PBS group, the TNC protein levels in the livers from mice in the CCl_4_ + TNC-CAR-Ms group were significantly lower (Fig. 4b). Similar results were observed by IHC and IF staining for TNC (Fig. 4c; Additional file 1: Fig. S5b). No significant changes in *Tnc* mRNA expression were observed after Mock-CAR-Ms or TNC-CAR-Ms treatment (Additional file 1: Fig. S5c). IF staining also showed that livers from the CCl_4_ + PBS group exhibited apoptotic cell death, as verified by terminal deoxynucleotidyl transferase-mediated dUTP nick-end labeling (TUNEL) staining, and increased levels of a damage-related proliferation marker, as verified by Ki67 staining. However, compared with those in the CCl_4_ + PBS group, the above characteristics of cell death and proliferation were reduced in the livers of the CCl_4_ + TNC-CAR-Ms group but not in those of the CCl_4_ + Mock-CAR-Ms group (Additional file 1: Fig. S5d). IHC staining for collagen I, a marker of activated HSCs, further indicated that TNC-CAR-Ms could reduce the number of activated HSCs (Additional file 1: Fig. S5e). In addition, IF staining revealed that the TNC protein colocalized with α-SMA^+^ cells in the livers of mice in the CCl_4_ + PBS group (Fig. 4d). While no significant differences were detected between the CCl_4_ + PBS and CCl_4_ + Mock-CAR-Ms groups, the number of TNC-positive α-SMA^+^ cells was significantly lower in livers from mice in the CCl_4_ + TNC-CAR-Ms group than in those from the CCl_4_ + PBS group (Fig. 4d), suggesting that TNC-positive HSCs were eliminated by TNC-CAR-Ms in the mouse liver after cell therapy. Taken together, these findings suggest that cell therapy with reprogrammed TNC-CAR-Ms delays liver fibrosis by increasing the degradation of the matrix protein TNC.

### ECM organization and cytokine-mediated signaling pathways participate in the antifibrotic mechanisms of TNC-CAR-Ms

**Fig. 5:**
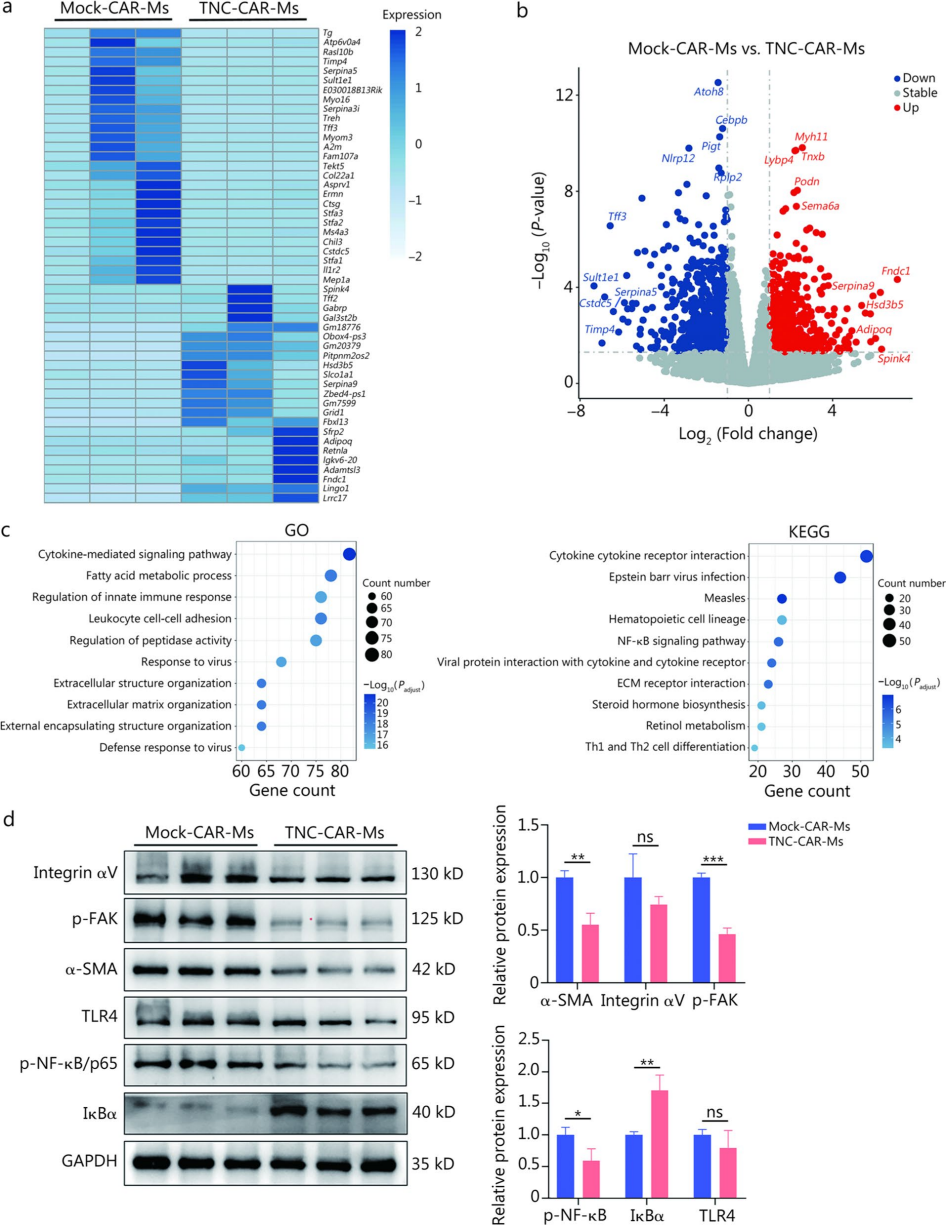
RNA sequencing analysis of liver tissues from Mock-CAR-Ms and TNC-CAR-Ms mice. **a** Heatmap showing the results for differentially expressed genes (DEGs) identified by RNA sequencing in the Mock-CAR-Ms and TNC-CAR-Ms groups. **b** Volcano plot of DEGs identified by RNA sequencing in the Mock-CAR-Ms and TNC-CAR-Ms groups, where upregulated genes are shown in red and downregulated genes are shown in blue. **c** The Gene Ontology (GO) and Kyoto Encyclopedia of Genes and Genomes (KEGG) enrichment analysis of the RNA sequencing data revealed the most significant biological processes that were changed by TNC-CAR-Ms compared with Mock-CAR-Ms. The bubble size represents the number of genes, and the color represents the *P-*value of the difference. **d** Levels of the α-SMA, integrin αV, p-FAK, p-NF-κB, IκBα, and TLR4 proteins in liver tissues from the CCl_4_ + Mock-CAR-Ms and CCl_4_ + TNC-CAR-Ms mice were detected by Western blotting and quantitatively analyzed using Quantity One software. Data are presented as mean ± SD. ^*^P < 0.05, ^**^P < 0.01, ^***^P < 0.001, ns non-significant. Integrin αV integrin alpha V, p-FAK phosphorylated focal adhesion kinase, α-SMA α-smooth muscle actin, TLR4 Toll-like receptor 4, p-NF-κB/p65 phosphorylated nuclear factor kappa B-p65 subunit, IκBα inhibitor of kappa B alpha, GAPDH glyceraldehyde-3-phosphate dehydrogenase, TNC tenascin-C, CAR-Ms chimeric antigen receptor-macrophages

We next performed an RNA sequencing analysis of liver tissues collected from the Mock-CAR-Ms and TNC-CAR-Ms groups to characterize the global changes in the liver after TNC-CAR-Ms therapy. A total of 1390 differentially expressed genes, including 711 downregulated genes and 679 upregulated genes, were identified in the TNC-CAR-Ms group compared with the Mock-CAR-Ms group. Heatmaps and volcano plots show that the expression levels of representative genes, such as *Myh11*, *Ltbp4*, *Tnxb*, *Podn*, *Fndc1*, *Serpina9,* and *Spink4,* were significantly upregulated in the TNC-CAR-Ms group, whereas the expression levels of *Cebpb*, *Pigt*, *Nlrp12*, *Tff3*, *Sult1e1,* and *Timp4* were significantly downregulated (Fig. 5a, b).

Gene Ontology and Kyoto Encyclopedia of Genes and Genomes enrichment analyses revealed the most significant biological processes that were changed by TNC-CAR-Ms (Fig. 5c). Among the top 10 pathways significantly altered by TNC-CAR-Ms, at least 5 were associated with ECM organization and cytokine-mediated signaling, indicating the antifibrotic molecular mechanism of TNC-CAR-Ms (Fig. 5c). In particular, the heatmap analysis revealed that the expression of genes enriched in cytokine-mediated signaling pathways, such as *Cxcl1, Ccl2*, *Ccr1*, *Cxcr2*, *Ccl3*, and *Il1r1,* generally tended to be downregulated in the TNC-CAR-Ms group (Additional file 1: Fig. S6a). Furthermore, the expression of genes enriched in ECM organization, such as *Bcl3*, *Col22a1*, *Nfkb2*, *Itgb3*, and *Fscn1*, was decreased markedly in TNC-CAR-Ms-treated mice (Additional file 1: Fig. S6b).

The levels of proteins related to ECM organization and cytokine-mediated signaling in the liver tissues of Mock-CAR-Ms- and TNC-CAR-Ms-treated mice were also analyzed by Western blotting. Similar to the results from the *Tnc* KO mice, the expression levels of p-NF-κB and p-FAK protein were significantly decreased in liver tissues collected from TNC-CAR-Ms-treated mice (Fig. 5d). Compared with the Mock-CAR-Ms group, the upregulated IκBα expression and downregulated α-SMA protein levels were also observed in the TNC-CAR-Ms group. Notably, the expression of TNC receptors, including integrin αV and TLR4, which are upstream of the TLR4/NF-κB and integrin/FAK pathways, changed only slightly in the TNC-CAR-Ms group (*P* > 0.05, Fig. 5d). Furthermore, the protein levels of cytokines, including TNF-α, IL-1β, and IL-6, were significantly decreased in liver tissues collected from TNC-CAR-Ms-treated mice (Additional file 1: Fig. S6c). These findings suggest that ECM organization, represented by the integrin/FAK pathway, and cytokine-mediated signaling pathways, represented by the TLR4/NF-κB pathway, were the major molecular mechanisms involved in the antifibrotic effects of TNC-CAR-Ms.

### M2-polarized TNC-CAR-Ms enhanced fibrosis regression in mice

**Fig. 6:**
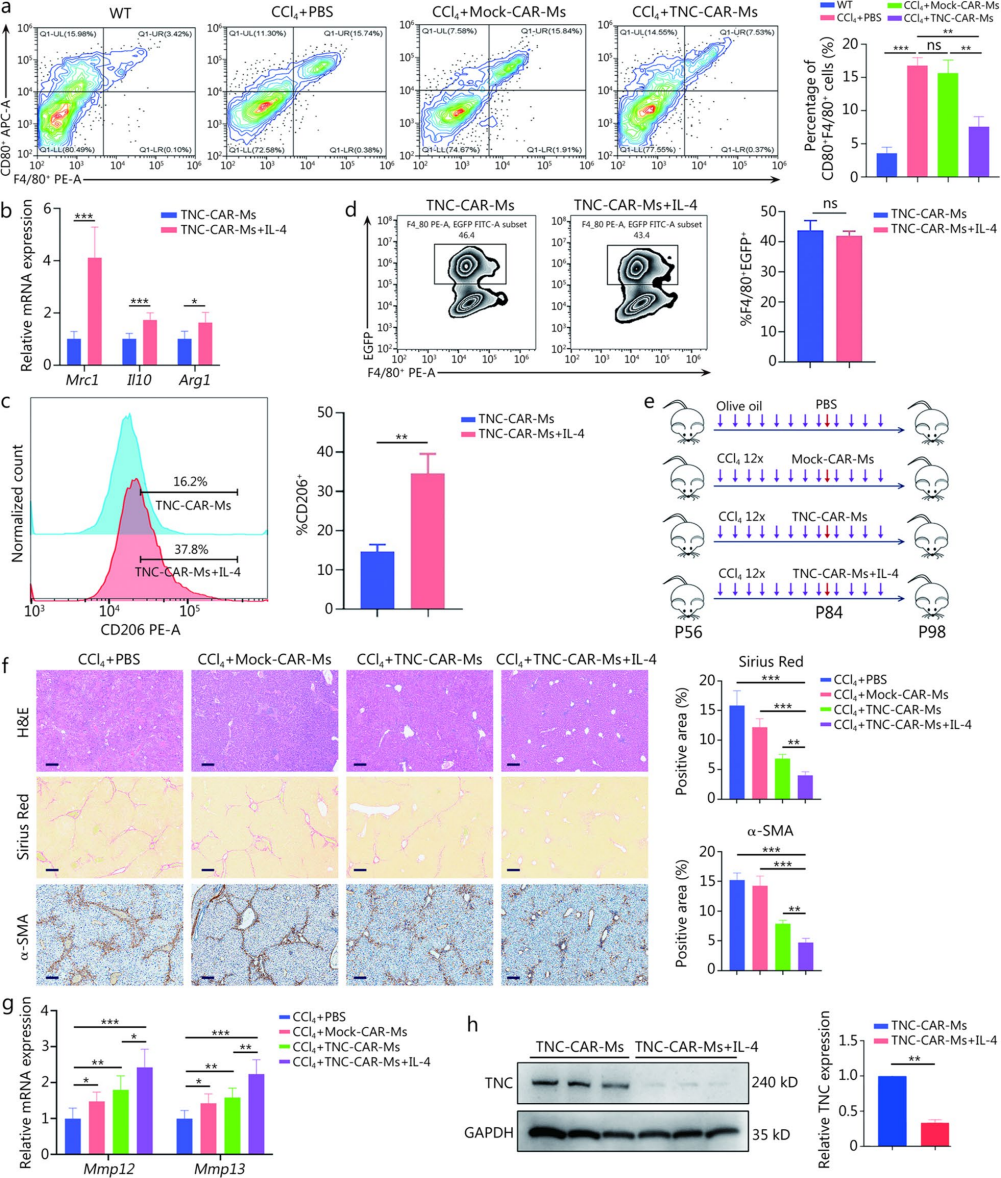
M2-polarized TNC-CAR-Ms enhanced fibrosis regression in mice. **a** The relative proportions of F4/80^+^CD80^+^ cells in liver tissues from WT, CCl_4_ + PBS, CCl_4_ + Mock-CAR-Ms, and CCl_4_ + TNC-CAR-Ms mice were detected by flow cytometry and quantitatively compared (*n* = 3). **b** RT-qPCR analysis of the expression levels of M2 polarization-related genes, including *Mrc1* (CD206), *Il10*, and *Arg1*, in TNC-CAR-Ms and IL-4-stimulated TNC-CAR-Ms. **c** The relative proportions of CD206^+^ cells among TNC-CAR-Ms and TNC-CAR-Ms + IL-4 cells were determined by flow cytometry and quantitatively compared. **d** Detection of phagocytic activity (F4/80^+^EGFP^+^ cells) in the coculture system using flow cytometry. **e** Experimental illustration of the effects of M2-polarized TNC-CAR-Ms on the liver fibrosis model. The CCl_4_-treated mice received PBS solution, Mock-CAR-Ms, TNC-CAR-Ms or IL4-stimulated TNC-CAR-Ms (i.v., 2 × 10^6^ cells/mouse) 24 h after the 8th CCl_4_ injection (*n* = 6). 12 × refers to the repeated injections of CCl_4_ for 12 times. P56, P84, and P98 indicate the mice’s age in postnatal days (day 56, day 84, and day 98, respectively). **f** Liver tissues from mice in the CCl_4_ + PBS, CCl_4_ + Mock-CAR-Ms, CCl_4_ + TNC-CAR-Ms, and CCl_4_ + TNC-CAR-Ms + IL-4 cell infusion groups were fixed with paraformaldehyde, embedded, sectioned, and subjected to H&E staining, Sirius Red staining, and α-SMA IHC staining. Quantification of Sirius Red staining and α-SMA IHC staining was performed using ImageJ software. Scale bar = 200 μm (H&E staining and Sirius Red staining) and 50 μm (α-SMA IHC staining). **g** RT-qPCR analysis of the expression of fibrinolytic genes, including *Mmp12* and *Mmp13*, in liver tissues from the CCl_4_ + PBS, CCl_4_ + Mock-CAR-Ms, CCl_4_ + TNC-CAR-Ms, and CCl_4_ + TNC-CAR-Ms + IL-4 cell infusion groups. **h** TNC protein expression in activated HSCs after TNC-CAR-Ms and TNC-CAR-Ms + IL-4 treatment was detected by Western blotting and quantitatively analyzed using Quantity One software. Data are presented as mean ± SD. ^*^P < 0.05, ^**^P < 0.01, ^***^P < 0.001, ns non-significant. CD80 cluster of differentiation 80, APC allophycocyanin, PE phycoerythrin, Mrc1 mannose receptor C-type 1, Il10 interleukin-10, Arg1 arginase 1, IL-4 interleukin-4, CD206 cluster of differentiation 206, EGFP enhanced green fluorescent protein, Mmp12 matrix metallopeptidase 12, Mmp13 matrix metallopeptidase 13, GAPDH glyceraldehyde-3-phosphate dehydrogenase, HSCs hepatic stellate cells, RT-qPCR reverse transcription quantitative polymerase chain reaction, α-SMA α-smooth muscle actin, IHC immunohistochemistry, TNC tenascin-C, CAR-Ms chimeric antigen receptor-macrophages

We further explored the effect of TNC-CAR-Ms on the immune cell population in the liver. TNC-CAR-Ms treatment significantly reduced the proportion of monocyte-derived macrophages (CD11b^+^F4/80^+^) (Additional file 1: Fig. S7a). In contrast, TNC-CAR-Ms treatment significantly increased the proportion of M2-polarized macrophages (CD206^+^F4/80^+^) (Additional file 1: Fig. S7b). RT-qPCR revealed that the expression of M2 marker genes, including *Mrc1* (CD206), *Il10,* and *Arg1,* was significantly increased in the TNC-CAR-Ms-treated group (Additional file 1: Fig. S7c). Compared with that in the control group, the proportion of CD80^+^F4/80^+^ macrophages in the TNC-CAR-Ms group was markedly reduced, suggesting that the TNC-CAR-Ms treatment robustly inhibited M1 macrophage polarization (Fig. 6a). This change might also result from the antagonism of TLR4/NF-κB signaling by the engineered TNC-CAR-Ms in the liver. Previous studies have suggested that M1-polarized macrophages exert a profibrogenic effect on the liver and that liver fibrosis can be ameliorated by induced pluripotent stem cell-derived macrophage populations, especially those of the M2 subtype [33–35]. We validated phenotypic plasticity by challenging TNC-CAR-transduced mouse macrophages with M2-inducing factors such as IL-4 in vitro. Upon stimulation with IL-4-conditioned media, the expression of canonical M2 marker genes such as *Mrc1* (CD206), *Il10*, and *Arg1* increased in TNC-CAR-engineered macrophages (Fig. 6b). M2-polarized CAR-Ms were also verified by flow cytometry (Fig. 6c). However, the in vitro killing and phagocytosis assays showed a similar effect on activated HSCs in the IL-4-stimulated and unstimulated groups (Fig. 6d; Additional file 1: Fig. S7d). In the CCl_4_-induced model, we confirmed that IL-4-stimulated TNC-CAR-Ms exhibited significantly increased antifibrotic activity, as indicated by the decreases in the intensity of Sirius Red staining and α-SMA IHC staining (Fig. 6e, f). RT-qPCR revealed that the expression of fibrinolysis-associated genes, such as *Mmp12* and *Mmp13,* was significantly increased in the M2-polarized TNC-CAR-Ms + IL-4 treatment group both in vitro and in vivo (Fig. 6g; Additional file 1: Fig. S7e). Furthermore, the results of in vitro coculture experiments showed that IL-4-stimulated TNC-CAR-Ms greatly accelerated the degradation of the TNC protein in activated HSCs (Fig. 6h). Taken together, the results revealed that the infusion of M2-polarized CAR-Ms significantly expedited fibrosis resolution, whereas the coculture of these cells with HSCs resulted in no obvious changes in the killing effect. These results indicate that the killing and phagocytic activities of macrophages are not the sole antifibrotic mechanisms of TNC-CAR-Ms, suggesting that other unknown molecular or cellular mechanisms may be involved in the antifibrotic functions of TNC-CAR-Ms therapy.

### Involvement of liver CD8^+^ T cells in TNC-CAR-Ms therapy and fibrosis resolution

**Fig. 7:**
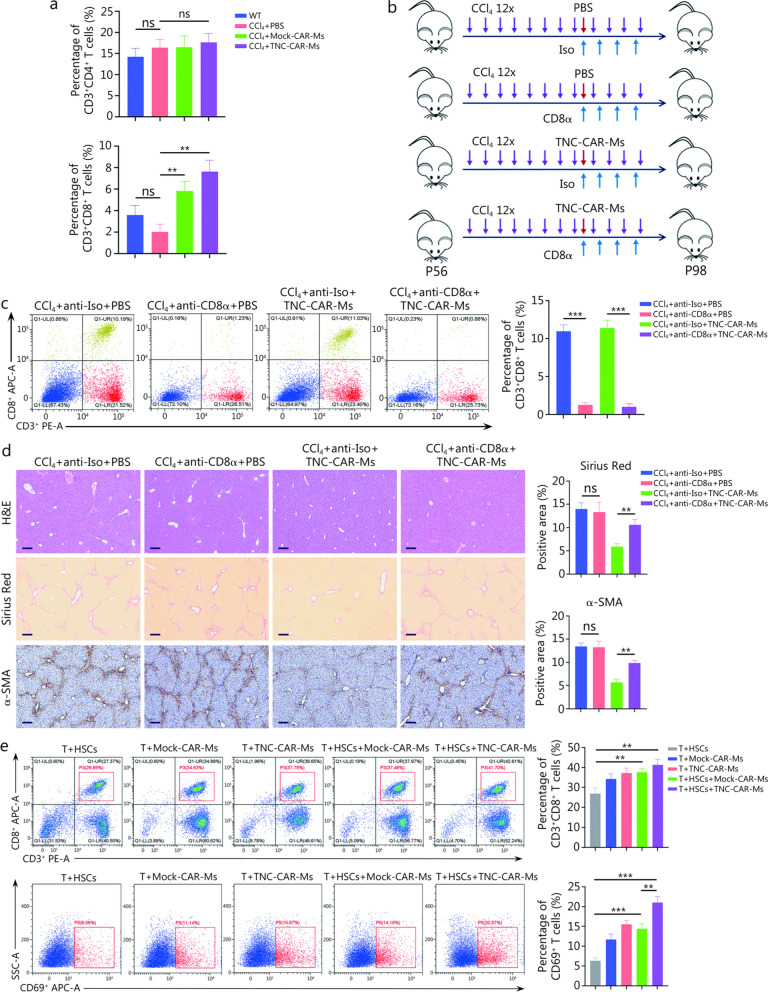
TNC-CAR-Ms exerted antifibrotic effects in a CD8^+^ T cell-dependent manner. **a** The relative proportions of CD3^+^CD4^+^ and CD3^+^CD8^+^ cells in liver tissues from WT, CCl_4_ + PBS, CCl_4_ + Mock-CAR-Ms, and CCl_4_ + TNC-CAR-Ms mice were determined by flow cytometry and quantitatively compared (*n* = 3). **b** Experimental illustration of the effect of CD8α antibody blockade on the antifibrotic effect of TNC-CAR-Ms on mice. The CCl_4_-treated mice received monoclonal CD8α antibody (anti-CD8α) or isotype control IgG antibody (anti-Iso) treatment every 3 d (i.p., 1 mg/kg), starting 1 d before the PBS and TNC-CAR-M (i.v., 2 × 10^6^ cells/mouse) infusions for 2 weeks (*n* = 6). The 12 × refers to the repeated injections of CCl_4_ for 12 times. P56 and P98 indicate the mice’s age in postnatal days (day 56 and day 98, respectively). **c** The relative proportions of CD3^+^CD8^+^ cells in the spleens of CCl_4_ + anti-Iso + PBS, CCl_4_ + anti-CD8α + PBS, CCl_4_ + anti-Iso + TNC-CAR-Ms, and CCl_4_ + anti-CD8α + TNC-CAR-Ms mice were determined by flow cytometry and quantitatively compared (*n* = 3). **d** Liver tissues from CCl_4_ + anti-Iso + PBS, CCl_4_ + anti-CD8α + PBS, CCl_4_ + anti-Iso + TNC-CAR-Ms, and CCl_4_ + anti-CD8α + TNC-CAR-M mice were fixed with paraformaldehyde, embedded, sectioned, and subjected to H&E staining, Sirius Red staining, and α-SMA IHC staining. Quantification of Sirius Red staining and α-SMA IHC staining was performed using ImageJ software. Scale bar = 200 μm (H&E staining and Sirius Red staining) and 50 μm (α-SMA IHC staining). **e** The relative proportions of CD3^+^CD8^+^ and CD69^+^ cells from the cocultured cell system, which consisted of CD3^+^ cells, HSCs, and CAR-Ms, were detected by flow cytometry and quantitatively compared (*n* = 3). Data are presented as mean ± SD. ^**^P < 0.01, ^***^P < 0.001, ns non-significant. CD3 cluster of differentiation 3, CD4 cluster of differentiation 4, CD8 cluster of differentiation 8, Iso isotype control, APC allophycocyanin, PE phycoerythrin, H&E hematoxylin and eosin, α-SMA α-smooth muscle actin, HSCs hepatic stellate cells, CD69 cluster of differentiation 69, SSC-A side scatter area, IHC immunohistochemistry, TNC tenascin-C, CAR-Ms chimeric antigen receptor-macrophages

We also explored the effect of TNC-CAR-Ms on the conventional T cell population in the liver. The flow cytometry results showed that compared with the CCl_4_ + PBS group, the CCl_4_ + Mock-CAR-Ms- and CCl_4_ + TNC-CAR-Ms-treated groups exhibited considerably increased proportions of the CD3^+^CD8^+^ immune cell population, but no significant change in the CD3^+^CD4^+^ cell population was observed (Fig. 7a; Additional file 1: Fig. S8a). Recent progress in studies of the adaptive immune system has clarified the role of CD8^+^ T cells in defending against viral infections and inflammatory diseases and in antitumor immunity [36–38]. Given that CD8^+^ T cells have multiple biological functions, we next determined whether the depletion of CD8^+^ T cells obstructs the antifibrotic effect of TNC-CAR-Ms. In CCl_4_-induced models, a mouse monoclonal anti-CD8α antibody and its isotype control antibody were intraperitoneally administered to mice before they were treated with TNC-CAR-Ms (Fig. 7b). As expected, the neutralizing anti-CD8α antibody resulted in a dramatic decrease in the percentage of CD8^+^ T cells in the mouse spleen (Fig. 7c). The results of IHC staining for CD8α in the liver also confirmed the depletion of CD8^+^ T cells in the mice (Additional file 1: Fig. S8b). Smaller scar sizes and less fibrotic tissue were detected by Sirius Red staining and α-SMA IHC staining in the livers collected from mice in the anti-Iso + TNC-CAR-Ms group, however, these parameters were significantly increased in the livers of mice from the anti-Iso + TNC-CAR-Ms group (Fig. 7d). In comparison, mice in the anti-CD8α + PBS group showed comparable degrees of liver fibrosis to those in the anti-Iso + PBS group (Fig. 7d). In addition, our in vitro results showed that the proportions of CD3^+^CD8^+^ and CD69^+^ cells increased after the coculture of HSCs, TNC-CAR-Ms, and T cells, indicating that CAR-Ms induce T cell activation through the presentation of the TNC antigen to T cells (Fig. 7e). The increased proportion of CD25^+^ cells observed after the coculture of HSCs, TNC-CAR-Ms, and T cells further suggested that TNC-CAR-Ms induce CD8^+^ T cell activation (Additional file 1: Fig. S8c). These results collectively reinforced the vital role of CD8^+^ T cells in the resolution of liver fibrosis by TNC-CAR-Ms therapy.

## Discussion

**Fig. 8:**
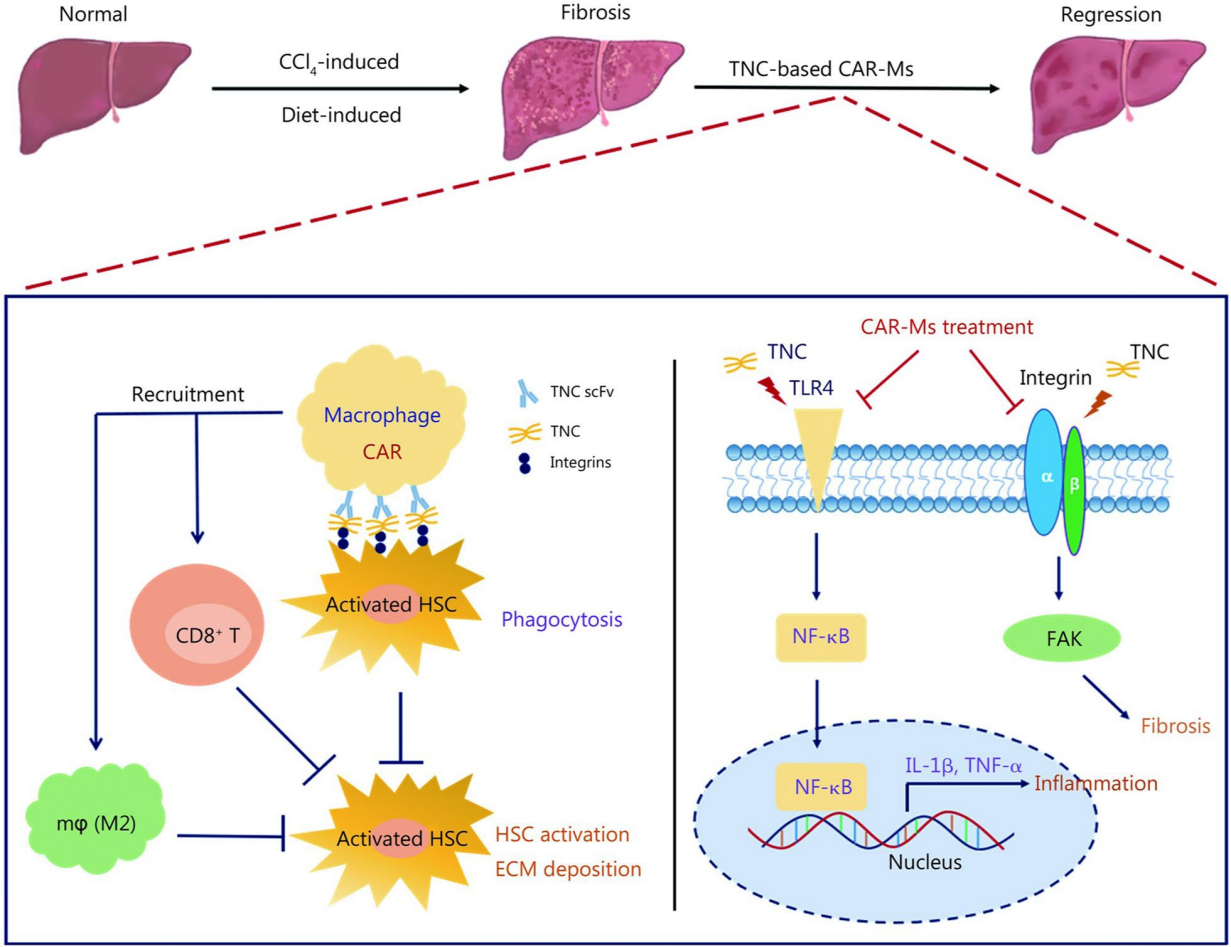
Diagram of the proposed mechanism of TNC-engineered CAR-Ms. At the cellular level, on one hand, the TNC-CAR-Ms harboring TNC scFv specifically recognize the TNC protein on the surface of HSCs to engulf activated HSCs, thereby inhibiting HSC activation and ECM deposition. On the other hand, TNC-CAR-Ms can also indirectly inhibit HSC activation and ECM deposition by recruiting CD8^+^ T cells and M2-polarized macrophages from the liver immune microenvironment. At the molecular level, TNC-CAR-Ms treatment can effectively prevent the activity of the TLR4/NF-κB inflammatory signaling pathway to reduce the expression of inflammatory factors IL-1β and TNF-α, and simultaneously inhibit Integrin-mediated FAK activation to prevent the fibrotic process. scFv single-chain fragment variable, CD8 cluster of differentiation 8, HSC hepatic stellate cell, ECM extracellular matrix, TLR4 Toll-like receptor 4, NF-κB nuclear factor kappa-B, FAK focal adhesion kinase, IL-1β interleukin-1β, TNF-α tumor necrosis factor-α, TNC tenascin-C, CAR-Ms chimeric antigen receptor-macrophages

In this study, we evaluated whether TNC-CAR-Ms can alleviate liver fibrosis. Upon the generation of second-generation TNC-CAR-Ms, we demonstrated their ability to engulf TNC-overexpressing HSCs in vitro. Next, we intravenously administered TNC-CAR-Ms to mice for 2 weeks, subjected them to short- and long-term CCl_4_ treatment, and an MCD-induced NASH fibrosis model. We found that TNC-CAR-Ms significantly reduced liver fibrosis in CCl_4_-treated model mice. We also observed that TNC-CAR-Ms mainly infiltrated the liver 3 d after cell transfer and reduced hepatic TNC protein levels, and more importantly, TNC-CAR-Ms led to a reduction in TNC protein levels in activated HSCs. No TNC-CAR-Ms-associated toxicity was detected in non-liver organs at 2 weeks after cell therapy. In addition, the results showed that TNC-CAR-Ms significantly increased the infiltration of M2 macrophages (F4/80^+^CD206^+^) into the liver and that adoptive transfer of M2-polarized CAR-Ms further improved the antifibrotic effect on CCl_4_-treated model animals despite the lack of a significant increase in killing activity in vitro. Finally, the blockade of CD8^+^ T cells by a CD8α-specific antibody markedly impaired the antifibrotic effect of TNC-CAR-Ms therapy in mice. Overall, at the cellular level, TNC-CAR-Ms suppress HSC activation and ECM deposition through phagocytosis (at a 10:1 E/T ratio) and the recruitment of M2-polarized macrophages and CD8^+^ T cells, and at the molecular level, TNC-CAR-Ms alleviate liver fibrosis via the TLR4/NF-κB and integrin/FAK signaling pathways (Fig. 8). Our work provides more evidence for the efficacy of using engineered CAR-M technology in the treatment of liver fibrosis.

Adoptive CAR-T cell therapy has been approved by the Food and Drug Administration (FDA) for patients with relapsed/refractory hematological malignancies in the clinic [39]. Given the remarkable efficacy of CAR-T cell therapy in hematological cancers, this technology has been extended to treat various diseases, including solid tumors, aging, and fibrotic disorders [40, 41]. However, the clinical value of this therapy remains limited by several barriers, such as transport and infiltration limitations, immunosuppressive microenvironments, and adverse reactions associated with CAR-T cell therapy [42]. Recently, CAR-Ms have been developed as a supplementary scheme or an alternative treatment to CAR-T cells. In tumor therapy research, macrophages modified with CARs have shown specific phagocytosis and tumor clearance capabilities by targeting tumor antigens both in vitro and in vivo. These modified macrophages also resist immunosuppressive cytokines and induce cytotoxic T cell function through antigen presentation [32]. Lei et al. [43] designed a second-generation CAR-Ms system by fusing the CD3ζ sequence with the intracellular Toll/IL-1 receptor (TIR) domain of TLR4, and discovered that the CAR modification enables induced pluripotent stem cell-derived CAR-Ms to exhibit antigen-dependent M1 polarization characteristics, enabling not only the phagocytosis of tumor cells but also the regulation of the tumor microenvironment. In the field of cardiovascular diseases, Chuang et al. [44] developed anti-CD47 CAR-Ms and found that CAR-Ms exhibited significantly enhanced phagocytic activity equivalent to that of CD47 antibody blockade. Furthermore, the surface of CAR-Ms was modified with ROS-responsive therapeutic nanoparticles, revealing that CAR and nanoparticle-engineered macrophages could resist immunosuppressive inflammatory environments and significantly enhance macrophage-mediated clearance of cellular debris in an atherosclerosis model [44].

Recent phase I and phase II clinical trials have highlighted the clinical therapeutic prospects of macrophage therapy [45, 46]. In the field of hepatic fibrosis treatment, the development of novel antifibrotic strategies based on macrophage therapy is imperative. In a representative study, Dai et al. [29] designed and constructed urokinase-type plasminogen activator receptor-targeted CAR-Ms. Evidence from a hepatic fibrosis mouse model indicated that the adoptive transfer of urokinase-type plasminogen activator receptor-targeted CAR-Ms led to a significant reduction in hepatic fibrosis and the restoration of liver function. Notably, Mao et al. [30] developed fibroblast activation protein (FAP)-targeted CAR-Ms. The phagocytosis of FAP^+^ cells specifically by FAP-CAR-Ms was observed in vitro, and in vivo animal experiments also confirmed that an intravenous infusion of FAP-CAR-Ms could effectively ameliorate CCl_4_-induced liver fibrosis in mice, indicating its therapeutic potential in the treatment of liver fibrosis [30]. In this work, we utilized our previously established lentiviral CAR-T systems to design a novel CAR-Ms tool based on the ECM-associated antigen TNC and administered it to hepatic fibrosis model mice. The results showed that intravenously administered TNC-CAR-Ms could mitigate liver tissue damage and fibrosis and reduce collagen deposition and α-SMA expression with no significant side effects on other organs. Fluorescent tracing techniques confirmed that TNC-CAR-Ms primarily accumulated in the liver, significantly inhibiting the expression of the TNC protein in the liver tissue. Moreover, TNC-CAR-Ms modulated the liver immune microenvironment, reducing the infiltration of proinflammatory M1 macrophages in liver tissues and inducing the liver residence of M2-polarized macrophages and CD8^+^ T cells. Whether CD8⁺ T cells mediate fibrosis resolution via cytotoxicity, cytokine modulation, or by facilitating further immune cell recruitment needs to be further explored. Therefore, the development of TNC as a novel CAR-Ms therapeutic target holds promise for further development and breakthroughs in macrophage therapy for hepatic fibrosis.

TNC-CAR-Ms represent a promising alternative for treating liver diseases in contrast to TNC-CAR-T cell therapy. Given the significant population of liver fibrosis/cirrhosis patients who may benefit from cell therapies, CAR-Ms represent a potentially more cost-effective option, as they enable standardized, large-batch production processes. However, a direct comparison of the efficacy of TNC-CAR-T cells and TNC-CAR-Ms in the context of liver fibrosis has yet to be rigorously determined.

TNC is highly expressed in various liver diseases, including chronic hepatitis, liver fibrosis, hepatic ischemia/reperfusion injury, and hepatocellular carcinoma [19, 47–49]. Using the CCl_4_-induced model, we found that TNC overexpression in the livers of CCl_4_-induced mice paralleled fibrosis progression, while loss of TNC expression in *Tnc* KO mice largely attenuated liver fibrosis. Moreover, the induction of TNC expression was observed in activated HSCs treated with the profibrogenic factor TGF-β. Previous studies have documented the efficacy of TNC-CAR-T cells in eliminating solid tumors such as breast cancer and brain tumors [20, 50]. Taken together, the results of our work indicate that TNC-CAR-Ms effectively resolve fibrosis by killing activated HSCs in the context of liver fibrosis. Furthermore, in addition to eliminating activated HSCs, enhancing M2-polarized macrophage infiltration and CD8^+^ T cell activation due to TNC degradation through the use of CAR technology has been validated for the treatment of liver fibrosis. Therefore, TNC has been identified as a multifunctional target for CAR-Ms since it not only can harness the phagocytic ability of CAR-Ms to accelerate the clearance of fibrotic and inflammatory HSCs in liver fibrosis but can also increase the infiltration of M2-polarized macrophages and the activation of CD8^+^ T cells in the hepatic immune microenvironment. At the molecular level, we speculate that the macrophages engineered with the TNC-scFv CAR competitively bind to the TNC protein, thereby reducing the availability of TNC to other cells and reducing the downstream activity of the TLR4/NF-κB and integrin/FAK signaling pathways, which can be considered a “molecular sponge effect”. The results revealed the cellular and molecular mechanisms underlying the ability of TNC-CAR-Ms to resolve liver fibrosis. Despite the ongoing research efforts focused on TNC, recent advancements in single-cell RNA sequencing technology have given us access to comprehensive data on cell type-specific gene expression patterns in the context of diverse liver diseases, discovering novel targets that may exhibit even greater potential than TNC for treating liver fibrosis associated with various liver diseases [51, 52].

## Conclusions

In summary, our study provides a proof-of-concept for the feasibility of alleviating liver fibrosis with TNC-CAR-Ms. Further investigations may help refine CAR constructs and identify other unique antigens that are expressed by activated HSCs or other cell types in the liver to improve their efficacy and safety properties before their translation to humans. Overall, our study presents a promising immunotherapeutic approach that involves the use of TNC-CAR-Ms to mitigate liver fibrosis and enhance liver function, and provides insights into a broader therapeutic horizon for a variety of liver diseases characterized by fibrotic manifestations.

## Supplementary Information

**Additional file 1.** Methods. **Fig. S1** TNC is highly expressed in livers from human fibrosis samples. **Fig. S2** TNC is highly expressed and mediates CCl4-induced liver fibrosis. **Fig. S3** Generation of TNC-CAR engineered macrophages and evaluation of phagocytic activity. **Fig. S4** TNC-CAR-Ms exhibited an antifibrotic effect in a variety of fibrosis mouse models. **Fig. S5** TNC-CAR-Ms migrate to the liver and reduce the TNC expression. **Fig. S6** RNA sequencing analysis of liver tissues from Mock-CAR-Ms and TNC-CAR-M mice.** Fig. S7** M2-polarized TNC-CAR macrophages enhanced the fibrosis regression in mice. **Fig. S8** TNC-CAR-Ms exhibited an anti-fibrosis effect in a CD8^+^ T-dependent manner. **Table S1** List of primers for RT-qPCR. **Table S2** Antibodies for flow cytometry. **Table S3** Antibodies for Western blotting and immunostaining. **Table S4** List of kits and enzymes used in the study.

## Data Availability

Supporting information is provided in the Additional file 1. Requests for any additional information and for materials should be addressed to the lead contact, Shuai-Shuai Zhang (zhangshuai2@scnu.edu.cn).

## Abbreviations

ALT: Alanine aminotransferase
APC: Allophycocyanin
α-SMA: α-smooth muscle actin
AST: Aspartate aminotransferase
BMDMs: Bone marrow-derived macrophages
CAR: Chimeric antigen receptor
CAR-Ms: Chimeric antigen receptor-macrophages
CCl_4_: Carbon tetrachloride
ECM: Extracellular matrix
EGFP: Enhanced green fluorescent protein
FAK: Focal adhesion kinase
FAP: Fibroblast activation protein
H&E: Hematoxylin and eosin
HSCs: Hepatic stellate cells
IF: Immunofluorescence
IHC: Immunohistochemistry
IL-4: Interleukin-4
KO: Knockout
MCD: Methionine choline deficient
MMPs: Matrix metalloproteinases
NASH: Nonalcoholic steatohepatitis
NF-κB: Nuclear factor kappa-B
PD-L1: Programmed cell death-ligand 1
PE: Phycoerythrin
RT-qPCR: Real-time quantitative polymerase chain reaction
scFv: Single-chain variable fragment
TGF-β: Transforming growth factor-β
TLR4: Toll-like receptor 4
TNC: Tenascin-C
WT: Wild-type
