# Supplementary material for: TNC-targeted CAR-macrophage therapy alleviates liver fibrosis in mice
Source: Mil Med Res. 2025 Nov 11;12:78. doi: 10.1186/s40779-025-00667-3 (PMC12604166; doi:10.1186/s40779-025-00667-3)
Supplement: Supplementary file 1 — Additional file 1. Methods. Fig. S1 TNC is highly expressed in livers from human fibrosis samples. Fig. S2 TNC is highly expressed and mediates CCl4-induced liver fibrosis. Fig. S3 Generation of TNC-CAR engineered macrophages and evaluation of phagocytic activity. Fig. S4 TNC-CAR-Ms exhibited an antifibrotic effect in a variety of fibrosis mouse models. Fig. S5 TNC-CAR-Ms migrate to the liver and reduce the TNC expression. Fig. S6 RNA sequencing analysis of liver tissues from Mock-CAR-Ms and TNC-CAR-M mice. Fig. S7 M2-polarized TNC-CAR macrophages enhanced the fibrosis regression in mice. Fig. S8 TNC-CAR-Ms exhibited an anti-fibrosis effect in a CD8+ T-dependent manner. Table S1 List of primers for RT-qPCR. Table S2 Antibodies for flow cytometry. Table S3 Antibodies for Western blotting and immunostaining. Table S4 List of kits and enzymes used in the study. [file 40779_2025_667_MOESM1_ESM.pdf]

Methods

Patient samples

The study protocols were conducted in accordance with the ethical guidelines of the Declaration of Helsinki Principles. Human fibrotic liver samples were collected from the Affiliated Hospital of Southwest Medical University with ethical approval from the institute (KY2023023).

Cell culture

RAW264.7 cell lines and immortalized bone marrow-derived macrophages (BMDMs) were obtained from Immocell Biological Technology Co., Ltd. (Xiamen, China). The HEK293T cell line was a gift from the laboratory of Xiaodong Ma. All cell lines were cultured in DMEM medium added with 10% fetal bovine serum (FBS). Mouse hepatic stellate cells (HSCs) were isolated by collagenase digestion according to gradient centrifugation, and then cultured in DMEM containing 10% FBS. To induce HSC activation, HSCs were serum starved overnight, and then treated with transforming growth factor (TGF)-β (20 ng/ml) for 24 h. The *EGFP* and luciferase genes were introduced into HSCs by lentiviral transduction for further bioluminescence analysis. Murine $CD3^+$ T cells were enriched from the spleen of mice using the Mouse T Cell Isolation Kit (19851, Stem Cell, Canada) in a 37 °C, 5% CO_2 incubator in complete RPMI 1640 medium (10% heat-inactivated FBS).

Lentiviral packaging

Lentiviral packaging was performed by seeding HEK293T cells in 10 cm dishes and co-transfecting them with the transfer vector, together with packaging plasmids (psPAX2) and envelope plasmids (pMD2.G). Transfection was carried out using PEI-MAX, following the manufacturer's established protocol to ensure efficient gene delivery. Forty-eight hours post-transfection, the culture supernatant containing the lentiviral particles was collected, and centrifugation was performed at 500× *g* for 10 min. Subsequently, the supernatant was carefully collected for passing through a 0.45 μm filter to remove cell debris. PEG8000 was added at a 1:4 ratio, and the mixture was placed at 4 °C for 24 h to facilitate concentration. Finally, centrifugation was performed at 2700× *g* for 30 min to obtain the viral pellet. The concentrated viral pellet was then resuspended in an appropriate volume of PBS and stored at –80 °C until further use.

Terminal deoxynucleotidyl transferase-mediated dUTP nick-end labeling (TUNEL) staining

TUNEL staining was carried out employing a fluorescence-based apoptosis detection kit (Alexa Fluor 640, Yeasen Biotech Co., Ltd., Shanghai, China) strictly according to the procedural guidelines outlined by the manufacturer.

Determination of serum biochemistry

Mice were sacrificed, and blood samples were obtained through retro-orbital bleeding. After standing at ambient temperature for 30 min, and subsequently centrifuging at 3500 rpm for 10 min, serum was collected. The assay kits for alanine aminotransferase (ALT), aspartate aminotransferase (AST), and albumin were utilized in strict accordance with the procedural guidelines provided by the manufacturer.

Determination of hydroxyproline in liver tissue

Mice liver tissues were collected under sterile conditions. A precisely weighed 90 mg portion of liver tissue was collected and homogenized in an appropriate volume of pre-chilled lysis buffer using a tissue homogenizer to ensure complete disruption of the sample. The resulting homogenate was subjected to acid hydrolysis with 6 mol/L HCl at 110 °C for 16 − 18 h to break down collagen into free hydroxyproline. After hydrolysis, the samples underwent neutralization before being subjected to subsequent analytical procedures in strict compliance with the guidelines provided by the manufacturer. The reaction mixture was incubated at 60 °C for 15 min to enable chromogenic substrate activation, ensuring optimal signal resolution for subsequent analysis. The optical density (OD) was measured at 550 nm using a microplate reader, and the hydroxyproline content was calculated based on a standard curve. The final hydroxyproline concentration was normalized to tissue weight (μg/mg liver tissue) to quantitatively assess collagen deposition and fibrosis levels.

Enzyme-linked immunosorbent assay (ELISA)

Serum and hepatic interleukin (IL)-1β, tumor necrosis factor (TNF)-α, and IL-6 concentrations were measured by ELISA in mice. Following 6 weeks of CCl_4 administration, blood was collected from WT and *Tnc* KO mice via retro-orbital bleeding. After allowing the samples to clot at room temperature for 30 min, they were centrifuged at 3500 rpm for 10 min at 4 °C to separate the serum. Commercially available ELISA kits were used according to the manufacturer's protocol (**Additional file 1: Table S2**). In brief, samples and standards were added to 96-well plates pre-coated with capture antibodies and incubated at 37 °C for 2 h. After thorough washing, biotinylated detection antibodies were added, followed by incubation with horseradish peroxidase (HRP)-conjugated streptavidin. The colorimetric reaction was initiated with tetramethylbenzidine substrate and terminated using a stop solution. Absorbance at 450 nm was recorded using a microplate reader, and cytokine concentrations were determined by referencing the standard curve. All measurements were performed in duplicate to ensure reliability.

Hematoxylin and eosin (H&E) staining

Liver tissue sections were stained using an H&E staining kit. Following staining, the sections subsequently

underwent dehydration through ethanol and xylene, and mounted with neutral resin. After air-drying naturally, sections were analyzed by a microscope.

Sirius Red staining

Liver tissue sections underwent Sirius Red staining using a commercially available kit, following the manufacturer's standardized protocol. Post-staining, the collagen deposition was quantified via ImageJ software.

Immunohistochemistry (IHC) staining

IHC was performed to detect protein expression in tissue sections. Paraffin-embedded liver tissues were deparaffinized with xylene and rehydrated through an ethanol gradient. Antigen repair was conducted using citrate buffer. Endogenous peroxidase activity was blocked with 3% H_2O_2 for 10 min, followed by blocking with 5% bovine serum albumin (BSA) for half an hour. After that, the sections were then incubated with primary antibodies (α-SMA, 1:5000; Tnc, 1:200; Ki67, 1:500; collagen I, 1:200; CD8α, 1:2000) overnight at 4 °C (**Additional file 1: Table S3**). After washing with PBS, HRP-conjugated secondary antibodies were added and incubated with liver sections. Protein signals were visualized using a diaminobenzidine (DAB) substrate, and sections were counterstained with hematoxylin. Acquiring images by using a light microscope, and protein expression levels were analyzed at the same time.

Immunofluorescence (IF) staining

Paraffin-embedded liver tissue sections were deparaffinized with xylene and rehydrated through a graded ethanol series. Antigen repair was conducted using citrate buffer. Endogenous peroxidase activity was blocked with 3% H_2O_2 for 10 min, followed by blocking with 5% BSA for 30 min. After that, the sections were incubated with primary antibodies (α-SMA, 1:1000; Tnc, 1: 200) overnight at 4 °C. After washing with PBS, Cy3-conjugated or fluorescein isothiocyanate (FITC)-conjugated secondary antibodies were added and incubated with liver sections. Images were acquired and analyzed using a laser confocal microscope (Zeiss, Germany).

Real-time quantitative polymerase chain reaction (RT-qPCR)

Total RNA for RT-qPCR was extracted from cells and liver tissues using an RNA extraction kit, and then using the reverse transcription kit to complete the synthesis of cDNA. RT-qPCR was performed using SYBR Green dye-based detection. The reaction mixture contained cDNA template, gene-specific primers, and Superstar Universal SYBR Green Master Mix (CWBio, Cat: CW3360). Following the manufacturer's protocol, set the RT-qPCR program on the Real-Time PCR System (Bio-Rad, CFX CONNECT Real-Time

System). Relative gene expression levels were quantified using the $2^{-\Delta\Delta Ct}$ analytical methods, with β-actin serving as the endogenous reference control. A detailed sequence of primers involved in the study can be found in **Additional file 1: Table S4**.

Western blotting analysis

For Western blotting analysis, total protein was isolated and subjected to quantitative evaluation via the BCA protein assay. Protein samples were fractionated via SDS-PAGE and subsequently immobilized onto PVDF membranes through an efficient transfer process. The membranes were preincubated in a 5% non-fat milk solution for 1 h to effectively block nonspecific binding sites, followed by an overnight exposure to primary antibodies at 4 °C to facilitate optimal antigen recognition (**Additional file 1: Table S3**). The next day, the membranes were washed with TBST (10 mmol/L Tris-HCl, pH 7.4, 150 mmol/L NaCl, 0.1% Tween-20) buffer and incubated with HRP-conjugated secondary antibodies at room temperature for 1.5 h. Protein bands were visualized using an enhanced chemiluminescence detection system and imaged using a gel imaging system (Tanon-4600). Protein band intensity was systematically measured using Quantity One software, employing GAPDH as the reference control to ensure normalization and comparative analysis.

$CD8^+$ T cell neutralization assay

To evaluate the functional role of $CD8^+$ T cells in the experimental setting, a $CD8^+$ T cell neutralization assay was conducted. C57BL/6J mice were intraperitoneally administered with anti-CD8α monoclonal antibody (Selleck, A2102) or Isotype control IgG antibody at a dose of 1 mg/kg every 3 days, starting one day before TNC-CAR-Ms or PBS injection. The depletion efficiency was confirmed via flow cytometry analysis of the proportion of $CD8^+$ T cells in the spleen with anti-CD8α antibody. At the end of the experiment, mice were euthanized, and liver samples were collected for histological examination.

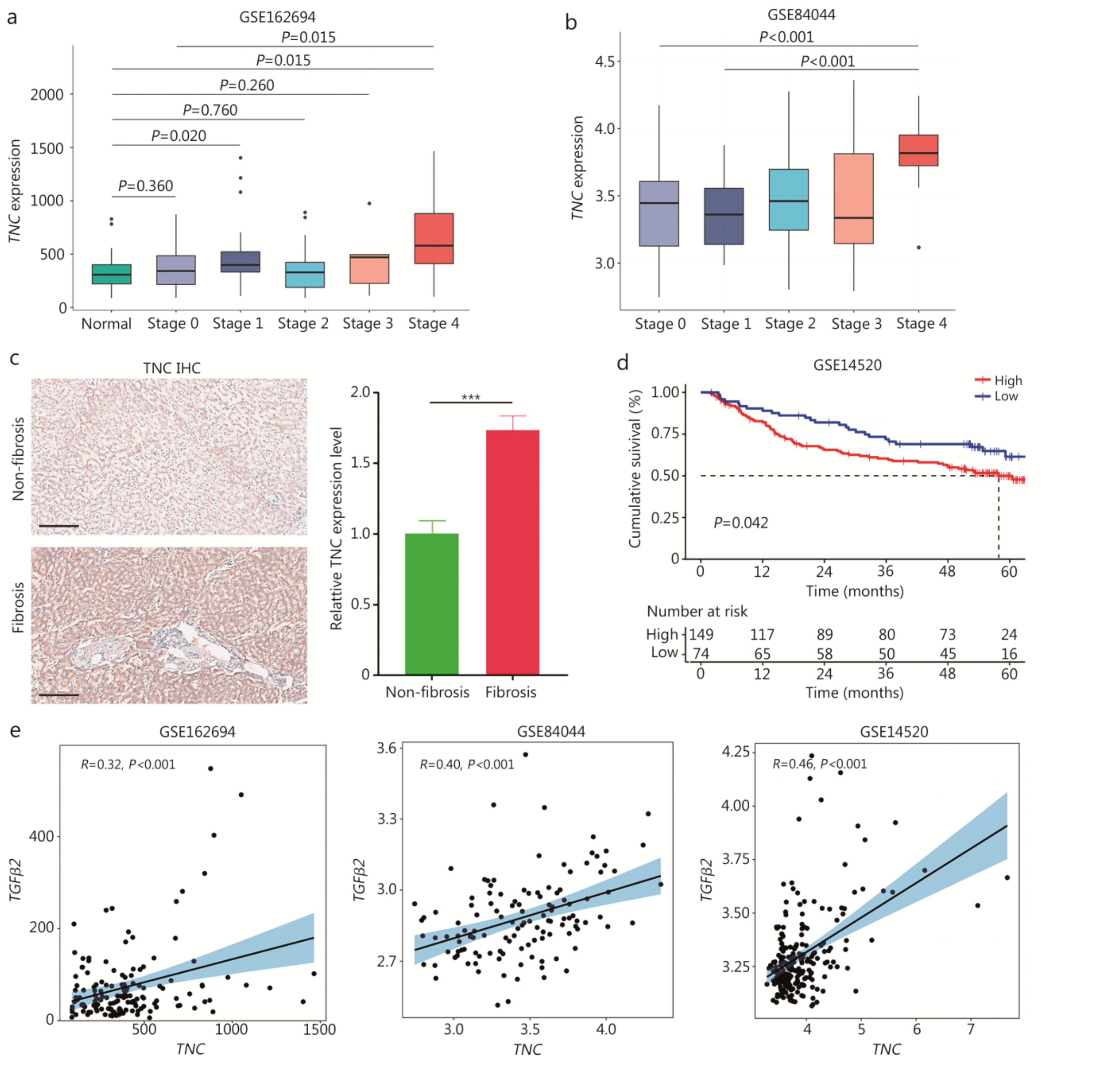

Fig. S1 TNC is highly expressed in livers from human fibrosis samples. **a, b** RNA-seq data analysis of the *TNC* expression in different stages of fibrosis patients and normal controls from the GEO dataset (GSE162694 and GSE84044). GSE162694: NASH patients with various fibrosis stages, normal controls, $n = 31$; stage 0, $n = 35$; stage 1, $n = 30$; stage 2, $n = 27$; stage 3, $n = 8$; stage 4, $n = 12$. GSE84044: HBV-related liver fibrosis patients, stage 0, $n = 43$; stage 1, $n = 20$; stage 2, $n = 33$; stage 3, $n = 18$; stage 4, $n = 10$. No normal controls in the GSE84044 dataset. **c** Representative immunohistochemical staining of TNC in tumor-adjacent fibrosis-free and fibrotic human livers, and its statistical summary. Scale bar = 200 μm. **d** Analysis of overall survival and TNC expression levels. TNC expression and follow-up

data, including a cohort of 223 HBV-related HCC patients with cirrhosis, were obtained through filtration of the GEO dataset (GSE14520). The cut-off of TNC was set by the optimal value. Based on the cut-off value, all cirrhosis patients were divided into high and low expression groups. **e** Pearson correlation analysis of *TNC* and *TGFβ2* expression in liver samples from GEO dataset (GSE162694, GSE84044, and GSE14520). Data are presented as mean ± SD. $^{***}P < 0.001$. GEO gene expression omnibus, IHC immunohistochemistry, RNA-Seq RNA sequencing, NASH non-alcoholic steatohepatitis, HCC hepatocellular carcinoma, TGFβ2 transforming growth factor-β2, TNC tenascin-C

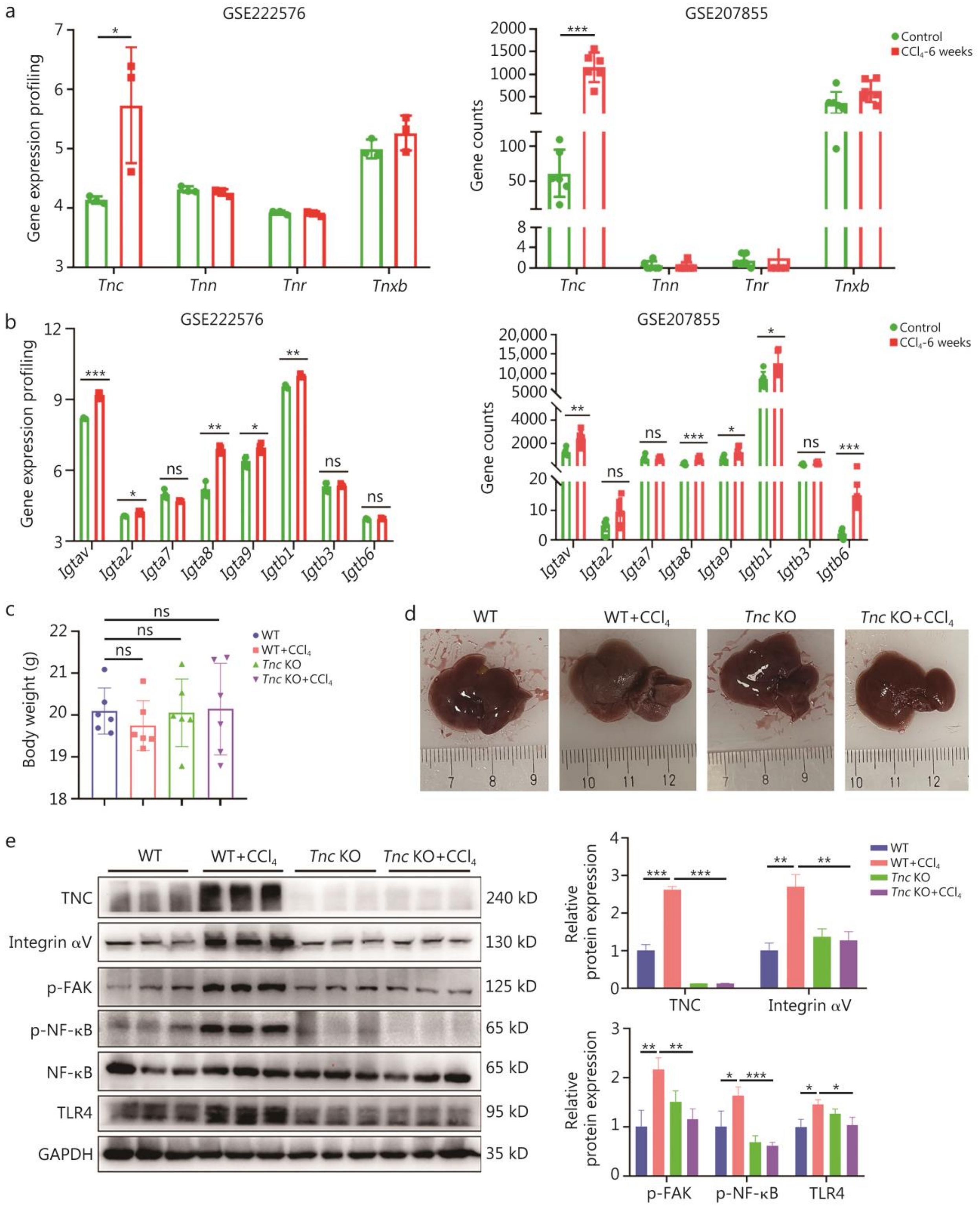

Fig. S2 TNC is highly expressed and mediates CCl4-induced liver fibrosis. **a, b** RNA-seq data analysis of the *Tnc* and its receptor encoding genes, integrins from microarray expression profile (GSE222576), and high-throughput sequencing expression profile (GSE207855). CCl4-6 weeks indicates mice received intraperitoneal injections of CCl4 for 6 weeks. **c**

Body weight of mice in WT, WT + CCl_4, *Tnc* KO, and *Tnc* KO + CCl_4 groups. **d** Representative liver images of mice in WT, WT + CCl_4, *Tnc* KO, and *Tnc* KO + CCl_4 groups. **e** Western blotting and quantification analysis of TNC, integrin αV, p-FAK, p-NF-κB, NF-κB, and TLR4 protein expression in liver tissues from WT, WT + CCl_4, *Tnc* KO, and *Tnc* KO + CCl_4 mice. Data are presented as mean ± SD. $^{*}P < 0.05$, $^{**}P < 0.01$, $^{***}P < 0.001$, ns non-significant. Tnn tenascin N, Tnr tenascin R, Tnxb tenascin XB, Itgav integrin subunit alpha V, Itga2 integrin subunit alpha 2, Itga7 integrin subunit alpha 7, Itga8 integrin subunit alpha 8, itga9 Integrin subunit alpha 9, Itgb1 integrin subunit beta 1, Itgb3 integrin subunit beta 3, Itgb6 integrin subunit beta 6, p-FAK phosphorylated focal adhesion kinase, p-NF-κB phosphorylated nuclear factor kappa-B, NF-κB nuclear factor kappa-B, TLR4 Toll-like receptor 4, GAPDH glyceraldehyde-3-phosphate dehydrogenase, TNC tenascin-C

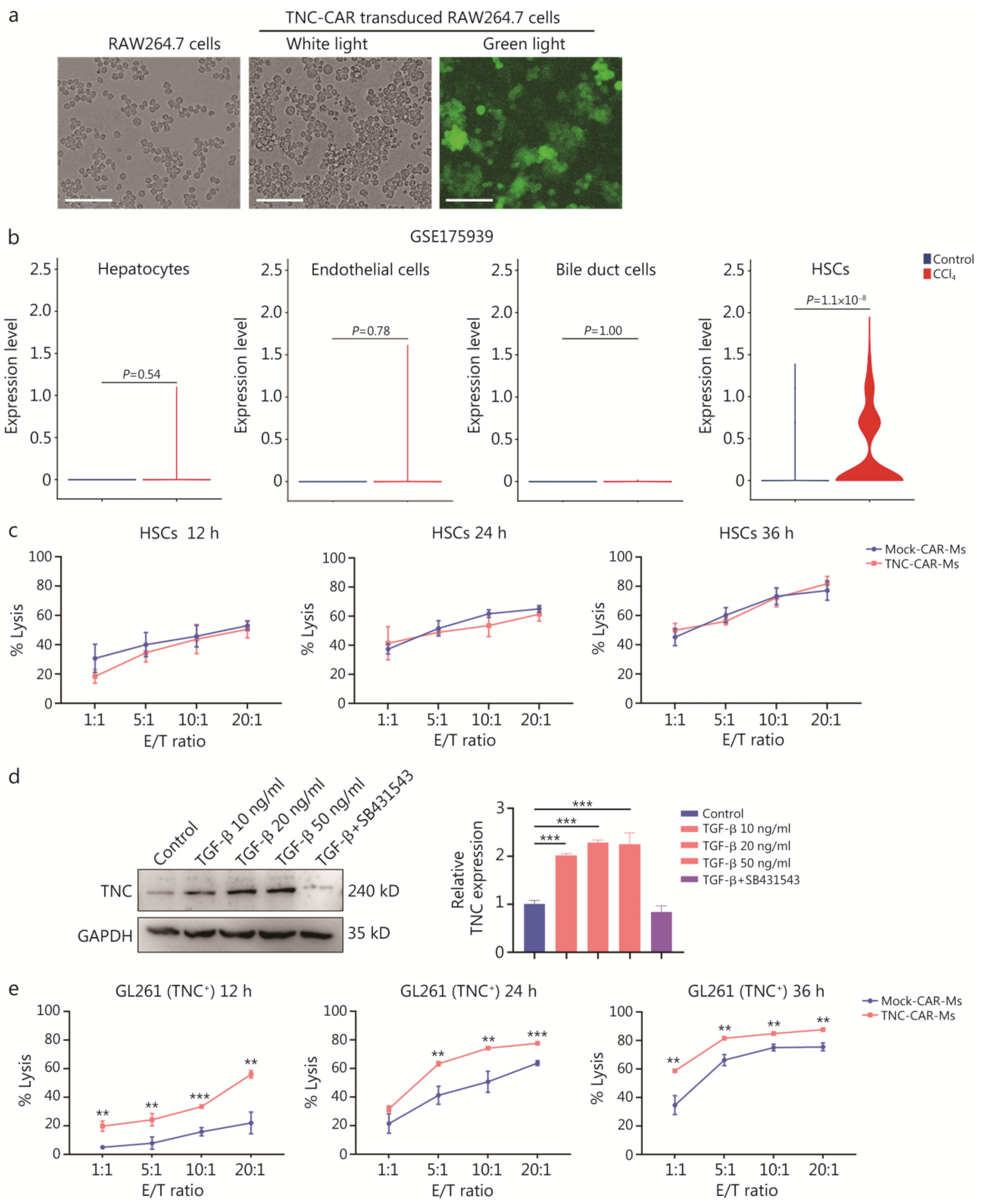

Fig. S3 Generation of TNC-CAR engineered macrophages and evaluation of phagocytic activity. **a** Microscopy images of TNC-CAR-transfected RAW264.7 cells and non-transfected cells, where the transfected TNC-CAR cells exhibited green fluorescence under a fluorescence microscope due to the presence of the EGFP protein. Scale bar = 100 μm. **b**

Violin plot analysis of single-cell RNA-sequencing (scRNA-seq) data of the *Tnc* gene in hepatocytes, endothelial cells, bile duct cells, and hepatic stellate cells (HSCs). **c** In vitro killing assay. Mouse HSCs (stably expressing luciferase) were co-cultured with Mock-CAR-Ms and TNC-CAR-Ms. After co-culture for 12, 24, and 36 h, cell viability and fluorescence intensity were assessed to evaluate the cytotoxicity of TNC-CAR-Ms and Mock-CAR-Ms against HSCs. **d** The expression of TNC protein in activated HSCs under TGF-β (0, 10, 20, 50 ng/ml) and TGF-β inhibitor (SB431542, 10 μmol/L) treatment was detected by Western blotting and quantitatively analyzed by Quantity One software. **e** In vitro killing assay. Murine GL261 tumor cells (stably expressing luciferase) were co-cultured with Mock-CAR-Ms and TNC-CAR-Ms. After co-culture for 12, 24, and 36 h, cell viability and fluorescence intensity were assessed to evaluate the cytotoxicity of TNC-CAR-Ms and Mock-CAR-Ms against GL261 cells. Data are presented as mean ± SD. $^{**}P < 0.01$, $^{***}P < 0.001$ vs. Mock-CAR-Ms. TGF-β transforming growth factor-β, GEO Gene Expression Omnibus, TNC tenascin-C, CAR-Ms chimeric antigen receptor-macrophages, E/T effector-to-target

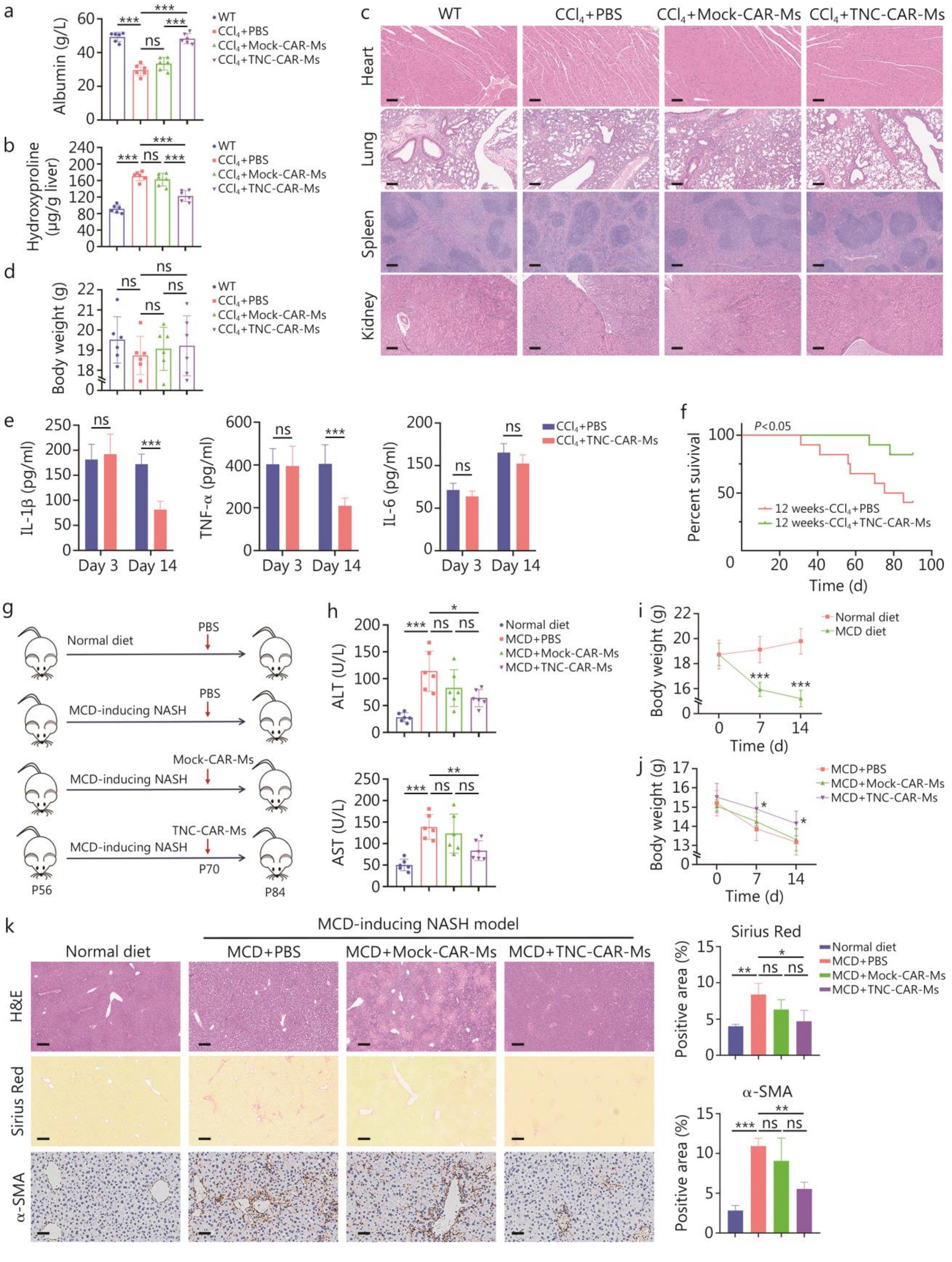

Fig. S4 TNC-CAR-Ms exhibited an antifibrotic effect in a variety of fibrosis mouse models. **a** Measurement of serum

albumin levels in mice from WT, CCl_4 + PBS, CCl_4 + Mock-CAR-Ms, and CCl_4 + TNC-CAR-Ms groups. **b** Detection of hydroxyproline content in liver tissues from WT, CCl_4 + PBS, CCl_4 + Mock-CAR-Ms, and CCl_4 + TNC-CAR-Ms mice. **c** H&E staining of mice organs, including heart, lungs, spleen, and kidneys from WT, CCl_4 + PBS, CCl_4 + Mock-CAR-Ms, and CCl_4 + TNC-CAR-Ms groups. Scale bar = 200 μm. **d** Body weight of mice in WT, CCl_4 + PBS, CCl_4 + Mock-CAR-Ms, and CCl_4 + TNC-CAR-Ms groups. **e** Detection of serum IL-1β, TNF-α, and IL-6 levels in CCl_4 + PBS and CCl_4 + TNC-CAR-Ms groups. **f** Analysis of survival rate in mice with cirrhosis (n = 12) after PBS infusion or TNC-CAR-Ms treatment for 90 d. **g** Experimental regime of MCD-inducing NASH fibrosis model in mice (n = 6). Normal diet mice were used as the negative control. MCD diet mice were divided into 3 groups. MCD mice from PBS, Mock-CAR-Ms, and TNC-CAR-Ms groups were given the MCD diet for 4 weeks and separately infused with PBS, Mock-CAR-Ms (2×10^6 cells in PBS), or TNC-CAR-Ms (2×10^6 cells in PBS) in the last 2 weeks. P56, P70, and P84 indicate the mice's age in postnatal days (day 56, day 70, and day 84, respectively). **h** Serum ALT and AST levels in different groups. At the endpoint, blood samples were collected from the mice, and serum was obtained by centrifugation. **i** Body weight of mice in the MCD diet group and the normal diet control group before cell infusion. $^{***}P < 0.001$ vs. Normal diet. **j** Body weight of MCD diet mice from PBS, Mock-CAR-Ms, and TNC-CAR-Ms groups after cell infusion. $^{*}P < 0.05$ vs. MCD + PBS. **k** Liver tissues from PBS, Mock-CAR-Ms, and TNC-CAR-Ms cell infusion and normal diet mice were fixed in paraformaldehyde, embedded in sections, and subjected to H&E staining, Sirius Red staining, and α-SMA IHC staining. Quantification analysis of Sirius Red staining and α-SMA IHC staining was performed using ImageJ software. Scale bar = 200 μm (H&E staining and Sirius Red staining) and 50 μm (α-SMA IHC staining). Data are presented as mean ± SD. $^{*}P < 0.05$, $^{**}P < 0.01$, $^{***}P < 0.001$, ns non-significant. IL-1β interleukin-1β, TNF-α tumor necrosis factor-α, IL-6 interleukin-6, i.v. intravenous, MCD methionine-choline deficient, NASH non-alcoholic steatohepatitis, AST aspartate aminotransferase, ALT alanine aminotransferase, H&E hematoxylin and eosin, α-SMA α-smooth muscle actin, IHC immunohistochemistry, TNC tenascin-C, CAR-Ms chimeric antigen receptor-macrophages

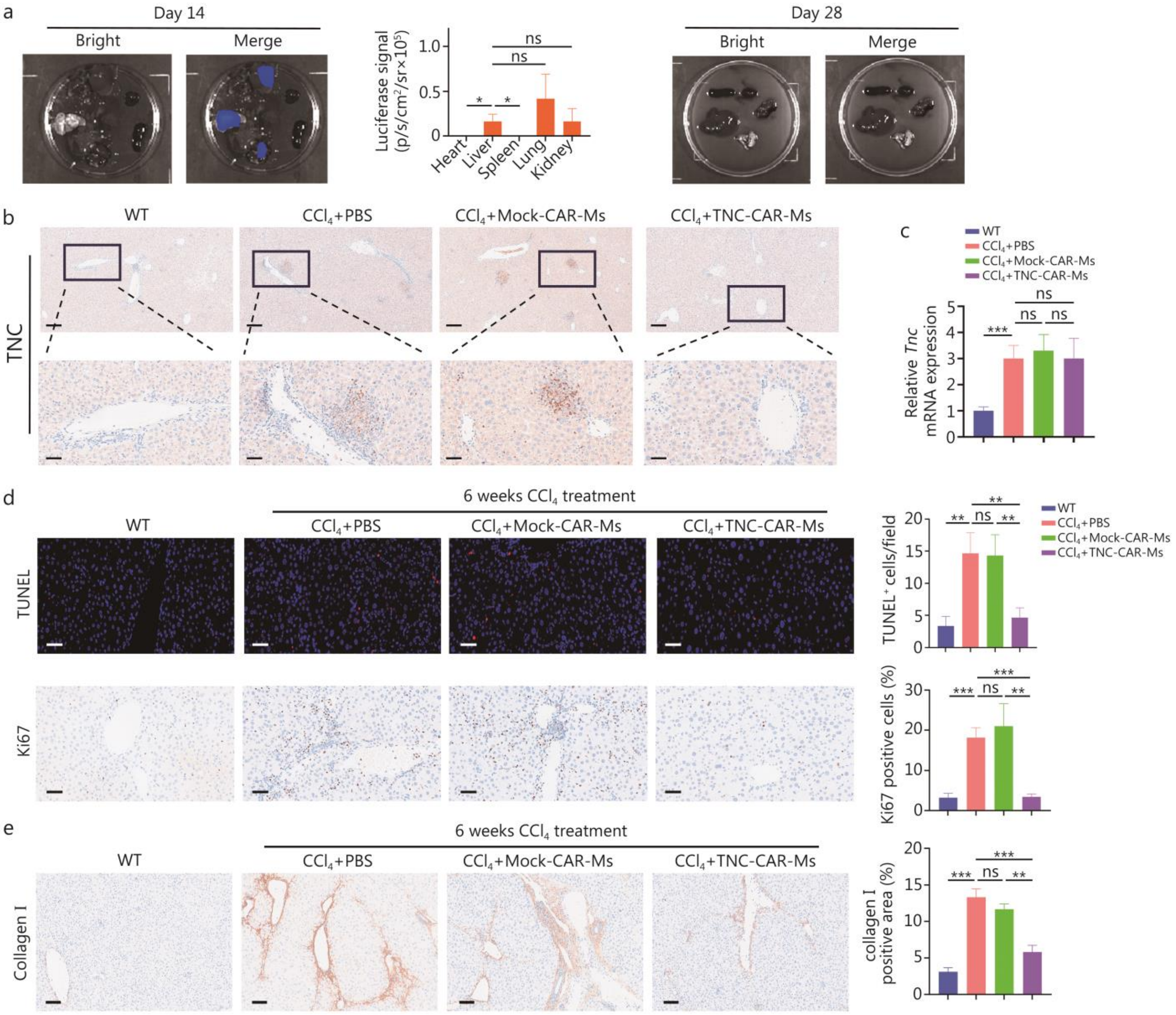

Fig. S5 TNC-CAR-Ms migrate to the liver and reduce the TNC expression. **a** Assessment of the biological distribution and transport of TNC-CAR-Ms in vivo. Day 14 and day 28 after TNC-CAR-Ms infusion, the mice were anesthetized and dissected. The distribution of luciferase-expressing TNC-CAR-Ms in the heart, liver, spleen, lung, and kidney tissues was monitored by Clinx-IVScope 8000 imaging system. The biological distribution of TNC-CAR-Ms at day 14 was quantitatively and statistically analyzed. **b** Liver tissues from WT, CCl_4 + PBS, CCl_4 + Mock-CAR-Ms, and CCl_4 + TNC-CAR-Ms mice were fixed with paraformaldehyde, embedded in sections, and subjected to TNC IHC staining analysis. Scale bar = 200 µm (upper) and 50 µm (lower). **c** RT-qPCR analysis of *Tnc* mRNA expression levels in liver tissues from WT, CCl_4 + PBS, CCl_4 + Mock-CAR-Ms, and CCl_4 + TNC-CAR-Ms mice. **d** Representative TUNEL apoptosis staining and immunohistochemical staining of Ki67 in livers from WT, CCl_4 + PBS, CCl_4 + Mock-CAR-Ms, and CCl_4 + TNC-

CAR-Ms mice, and its statistical summary (n = 3). Scale bar = 50 μm. **e** Representative immunohistochemical staining of collagen I in livers from WT, CCl_4 + PBS, CCl_4 + Mock-CAR-Ms, and CCl_4 + TNC-CAR-Ms mice, and its statistical analysis (n = 3). Scale bar = 100 μm. Data are presented as mean ± SD. $^{*}P < 0.05$, $^{**}P < 0.01$, $^{***}P < 0.001$, ns non-significant. IHC immunohistochemistry, RT-qPCR reverse transcription quantitative polymerase chain reaction, TUNEL terminal deoxynucleotidyl transferase-mediated dUTP nick-end labeling, TNC tenascin-C, CAR-Ms chimeric antigen receptor-macrophages

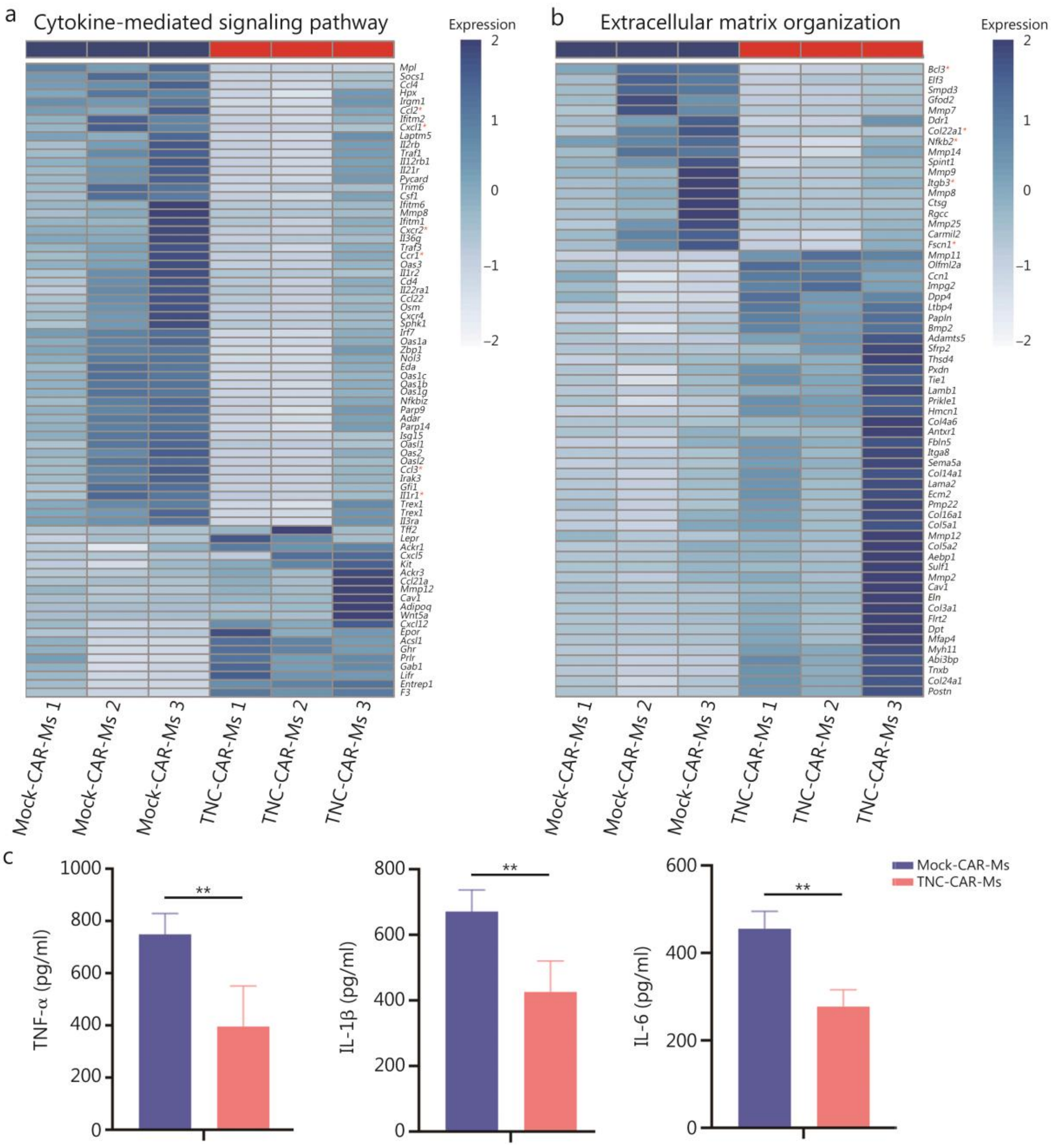

Fig. S6 RNA sequencing analysis of liver tissues from Mock-CAR-Ms and TNC-CAR-Ms mice. **a** Heatmap of gene clusters in cytokine-mediated signaling pathway enrichment analysis from RNA sequencing data in Mock-CAR-Ms and TNC-CAR-Ms groups. **b** Heatmap results of gene cluster in extracellular matrix organization enrichment analysis from RNA sequencing data in Mock-CAR-Ms and TNC-CAR-Ms groups. **c** Tumor necrosis factor-α (TNF-α), interleukin-1β (IL-1β), and interleukin-6 (IL-6) levels in liver tissues from TNC-CAR-Ms and Mock-CAR-Ms groups ($n = 6$). Data are presented as mean ± SD. $^{**}P < 0.01$. ELISA enzyme-linked immunosorbent assay, TNC tenascin-C, CAR-Ms chimeric antigen receptor-macrophages

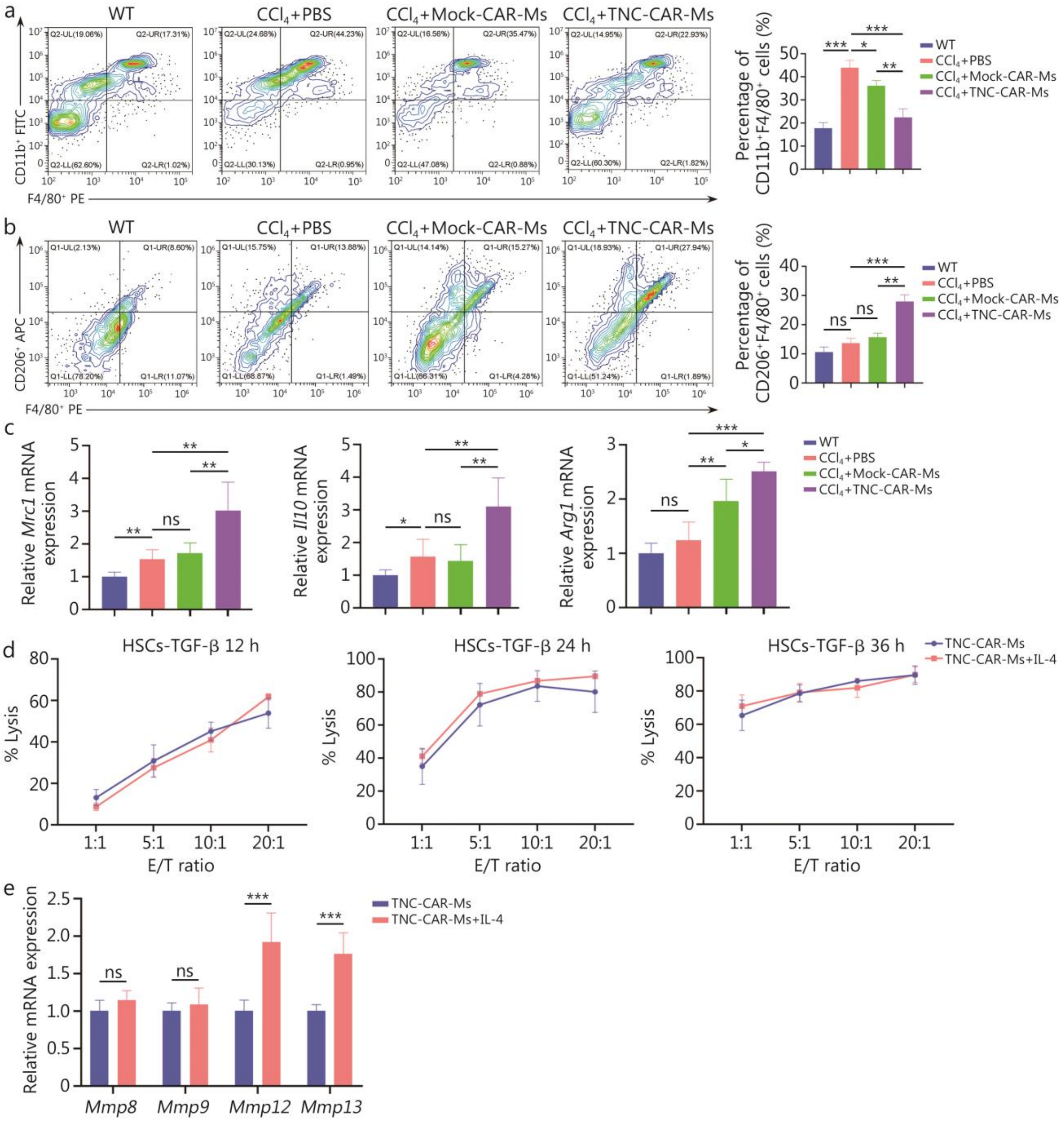

Fig. S7 M2-polarized TNC-CAR macrophages enhanced the fibrosis regression in mice. **a** The relative proportion of CD11b$^+$F4/80$^+$ cells in liver tissues from WT, CCl_4 + PBS, CCl_4 + Mock-CAR-Ms, and CCl_4 + TNC-CAR-Ms mice were detected by flow cytometry and quantitatively compared (n = 3). **b** The relative proportion of CD206$^+$F4/80$^+$ cells in liver tissues from WT, CCl_4 + PBS, CCl_4 + Mock-CAR-Ms, and CCl_4 + TNC-CAR-Ms mice were detected by flow cytometry and quantitatively compared (n = 3). **c** RT-qPCR analysis of M2 polarization genes including *Mrc1*, *Il10,* and *Arg1* mRNA expression levels in liver tissues from WT, CCl_4 + PBS, CCl_4 + Mock-CAR-Ms, and CCl_4 + TNC-CAR-Ms mice (n = 6). **d** In vitro killing assay. TGF-β-stimulated HSCs (stably expressing luciferase) were co-cultured with TNC-CAR-Ms and

TNC-CAR-Ms + IL4 cells. After co-culture for 12, 24, and 36 h, cell viability and fluorescence intensity were assessed to evaluate the cytotoxicity of TNC-CAR-Ms and TNC-CAR-Ms + IL4 cells against HSC cells. **e** RT-qPCR analysis of fibrinolysis-associated genes including *Mmp8, Mmp9, Mmp12,* and *Mmp13* expression in TNC-CAR-Ms and TNC-CAR-Ms + IL4 cells. Data are presented as mean ± SD. $^{*}P < 0.05$, $^{**}P < 0.01$, $^{***}P < 0.001$, ns non-significant. FITC fluorescein isothiocyanate, PE phycoerythrin, Mrc1 mannose receptor c-type 1, Il10 interleukin 10, Arg1 arginase 1, HSCs hepatic stellate cells, TGF-β transforming growth factor-β, Il4 interleukin 4, Mmp8 matrix metallopeptidase 8, Mmp9 matrix metallopeptidase 9, Mmp12 matrix metallopeptidase 12, Mmp13 matrix metallopeptidase 13, RT-qPCR reverse transcription quantitative polymerase chain reaction, TGF-β transforming growth factor-β, TNC tenascin-C, CAR-Ms chimeric antigen receptor-macrophages, E/T effector-to-target

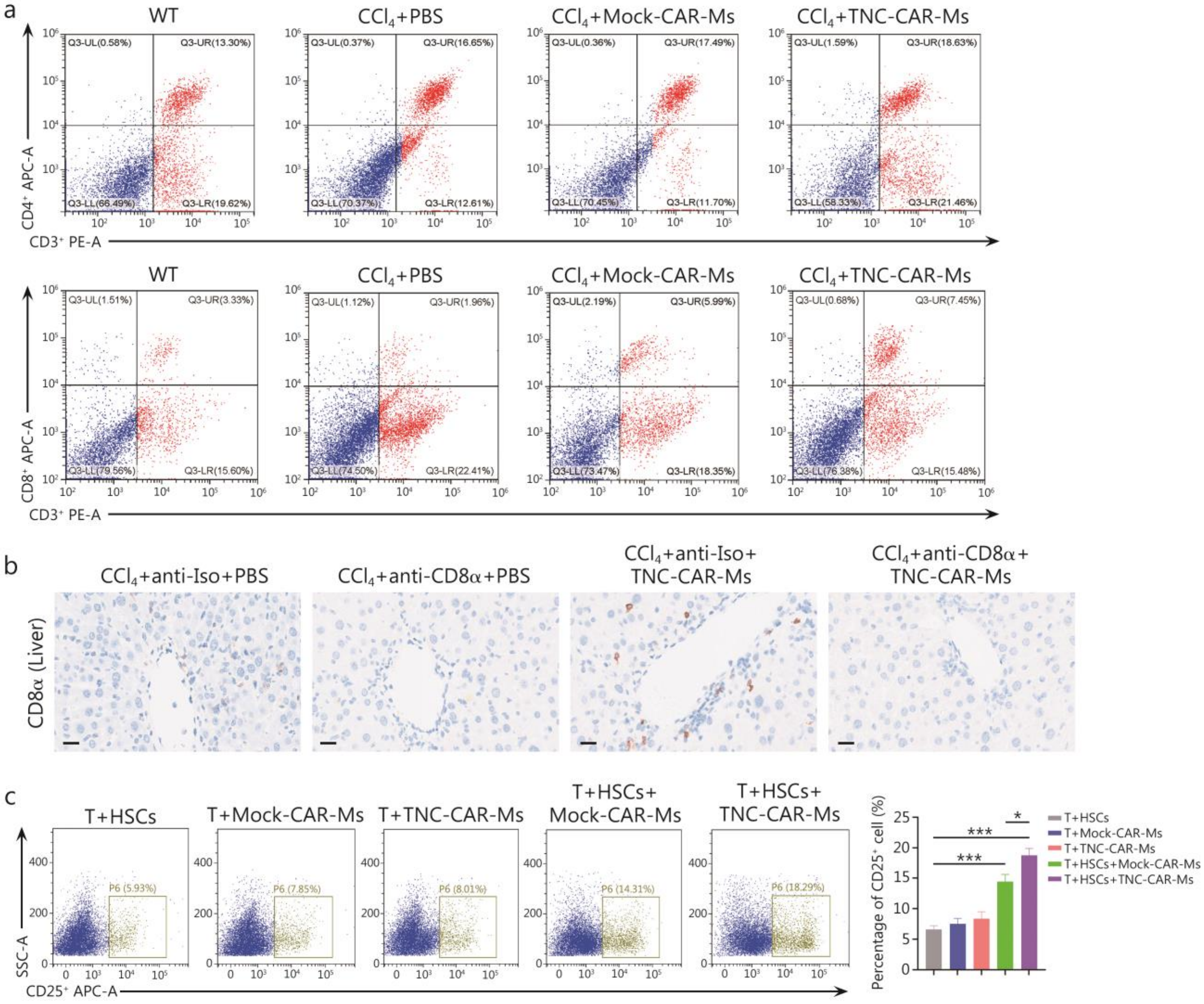

Fig. S8 TNC-CAR-Ms exhibited an anti-fibrosis effect in a $CD8^+$ T cell-dependent manner. **a** Representative flow cytometry images of $CD3^+CD4^+$ cells and $CD3^+CD8^+$ cells in liver tissues from WT, CCl_4 + PBS, CCl_4 + Mock-CAR-Ms, and CCl_4 + TNC-CAR-Ms mice. **b** Representative immunohistochemical staining of CD8α in livers from CCl_4 + anti-Iso + PBS, CCl_4 + anti-CD8α + PBS, CCl_4 + anti-Iso + TNC-CAR-Ms, and CCl_4 + anti-CD8α + TNC-CAR-Ms mice (n = 3). Scale bar = 25 μm. **c** Relative proportion of $CD25^+$ cells from the co-cultured cell system consisting of $CD3^+$ cells, HSCs, and CAR-macrophages was detected by flow cytometry and quantitatively compared (n = 3). Data are presented as mean ± SD. $^{*}P < 0.05$, $^{***}P < 0.001$. FITC fluorescein isothiocyanate, PE phycoerythrin, APC allophycocyanin, Iso isotype control, HSCs hepatic stellate cells, TNC tenascin-C, CAR-Ms chimeric antigen receptor-macrophages

Table S1 Antibodies for flow cytometry

Item name	Brand/Supplier	Catalog number
Biotin-Protein L	Genscript	M00097
Streptavidin PE Conjugate	Invitrogen	12-4317-87
CD11b Monoclonal Antibody	Invitrogen	11-0112-82
Anti-Mouse F4/80	BD Bioscience	565410
Anti-Mouse CD206	Biolegend	141707
CD80 Monoclonal Antibody	Invitrogen	17-0801-82
CD3 Monoclonal Antibody	Invitrogen	12-0032-82
Anti-Mouse CD8a	Biolegend	100712
Anti-Mouse CD4	Biolegend	100433

PE phycoerythrin, *CD11b* cluster of differentiation 11b, *CD206* cluster of differentiation 206, *CD80* cluster of differentiation 80, *CD3* cluster of differentiation 3, *CD8a* cluster of differentiation 8 alpha, *CD4* cluster of differentiation 4

Table S2 List of kits and enzymes used in the study

Item name	Brand/Supplier	Catalog number
RNA Extraction Kit	GOONIE	400-100
Reverse Transcription Kit	Yugong Biotech	EG15133S
qPCR Master Mix	CW Bio	CW3360
Plasmid Extraction Kit	CW Bio	CW2105S
2×Phanta Flash Master Mix	Vazyme	P520
In-Fusion HD Cloning kits	Takara Bio	639649
Aspartate Aminotransferase Kit	Nanjing Jiancheng	C010-3-1
Alanine Aminotransferase Kit	Nanjing Jiancheng	C009-3-1
Albumin Assay Kit	Nanjing Jiancheng	A028-2-1
Hydroxyproline assay kit	Nanjing Jiancheng	A030-2-1
Mouse IL-1β ELISA Kit	ABclonal	RK04878
Mouse IL-6 ELISA Kit	ABclonal	RK00008
Mouse TNF-α ELISA Kit	ABclonal	RK04875
TUNEL Apoptosis Detection Kit	Yeasen Biotech	40308ES20
Hematoxylin-Eosin Staining Kit	Yeasen Biotech	60524ES60
Sirius Red Staining Kit	Yeasen Biotech	60415ES50
EasySep Mouse T Cell Isolation Kit	Stemcell	19851

qPCR quantitative polymerase chain reaction, *IL-1β* interleukin-1β, *IL-6* interleukin-6, *TNF-α* tumor necrosis factor-α, *ELISA* enzyme-linked immunosorbent assay

Table S3 Antibodies for immunohistochemistry and Western blotting

**Item name**	**Brand/Supplier**	**Catalog number**
Tenascin C (TNC) Antibody	ABclonal	A18156
Integrin αV Antibody	ABclonal	A19071
p-FAK Antibody	ABclonal	AP1447
p-NF-κB Antibody	ABclonal	AP1528
NF-κB Antibody	ABclonal	A11160
TLR4 Antibody	ABclonal	A11226
GAPDH Monoclonal Antibody	Proteintech	60004-1-Ig
α-SMA Monoclonal Antibody	Proteintech	67735-1-Ig
Collagen Type I Monoclonal Antibody	Proteintech	66761-1-Ig
CD8a Recombinant Antibody	Proteintech	85977-4-RR
Ki67 Antibody	ABclonal	A20018
IκBα Antibody	ABclonal	A1187
HRP-labeled Goat anti-Rabbit	Yeasen	33101ES60
HRP-labeled Goat anti-Mouse	Yeasen	33201ES60
FITC-labeled Goat anti-Mouse	ABclonal	AS001
Cy3-labeled Goat anti-Rabbit	ABclonal	AS007

p-FAK phosphorylated focal adhesion kinase, *p-NF-κB* phosphorylated nuclear factor kappa-B, *NF-κB* nuclear factor kappa-B, *TLR4* Toll-like receptor 4, *GAPDH* glyceraldehyde-3-phosphate dehydrogenase, *α-SMA* α-smooth muscle actin, *CD8a* cluster of differentiation 8 alpha, *Ki67* Kiel-67, *IκBα* inhibitor of kappa B alpha, *HRP* horseradish peroxidase, *FITC* fluorescein isothiocyanate, *Cy3* cyanine 3

Table S4 Primers for RT-qPCR

**Gene**	**Primers**	**Sequence (5'-3')**
β-actin	Forward	ACCCGCCACCAGTTCGC
	Reverse	CACGATGGAGGGGAAGACG
Tnc	Forward	ACGGCTACCACAGAAGCTG
	Reverse	ATGGCTGTTGTTGCTATGGCA
Il-1β	Forward	CTGTGACTCGTGGGATGATG
	Reverse	GGGATTTTGTCGTTGCTTGT
Cxcl1	Forward	CTGGGATTCACCTCAAGAACATC
	Reverse	CAGGGTCAAGGCAAGCCTC
Il-10	Forward	CCTGCTCTTACTGGCTGGAG
	Reverse	TGTCCAGCTGGTCCTTCTTT
Acta2	Forward	GTTCAGTGGTGCCTCTGTCA
	Reverse	ACTGGGACGACATGGAAAAG
Col1a1	Forward	TAGGCCATTGTGTATGCAGC
	Reverse	ACATGTTCAGCTTTGTGGACC
Col2a1	Forward	AGCAGGTCCTTGGAAACCTT
	Reverse	AAGGAGTTTCATCTGGCCCT
Mmp8	Forward	CAGGGAGAAGCAGACATCAACA
	Reverse	GATTCCATTGGGTCCATCAAA
Mmp9	Forward	CCATGCACTGGGCTTAGATCA
	Reverse	GGCCTTGGGTCAGGCTTAGA
Mmp12	Forward	GCTGCTCCCATGAATGACAG
	Reverse	TGCCAGAGTTGAGTTGTCCA
Mmp13	Forward	TGATGATGAAACCTGGACAAGCA
	Reverse	GGTCCTTGGAGTGATCCAGACCTA
Arg1	Forward	AGACAGCAGAGGAGGTGAAGAG
	Reverse	CGAAGCAAGCCAAGGTTAAAGC
Mrc1	Forward	TTCAGCTATTGGACGCGAGG

Gene	Primers	Sequence (5'-3')
	Reverse	GAATCTGACACCCAGCGGAA
Ym1	Forward	CATTCAGTCAGTTATCAGATTCC
	Reverse	AGTGAGTAGCAGCCTTGG
Nos2	Forward	GCAGAGATTGGAGGCCTTGTG
	Reverse	GGGTTGTTGCTGAACTTCCAGTC
Tnf-α	Forward	CAGGAGGGAGAACAGAAACTCCA
	Reverse	CCTGGTTGGCTGCTTGCTT

Tnc tenascin C, *Il-1β* interleukin-1β, *Cxcl1* chemokine (C-X-C motif) ligand 1, *Il-10* interleukin-10, *Acta2* actin alpha 2, *Col1a1* collagen type I alpha 1 chain, *Col2a1* collagen type II alpha 1 chain, *Mmp8* matrix metallopeptidase 8, *Mmp9* matrix metallopeptidase 9, *Mmp12* matrix metallopeptidase 12, *Mmp13* matrix metallopeptidase 13, *Arg1* arginase 1, *Mrc1* mannose receptor C-type 1, *Ym1* chitinase-3-like protein 3, *Nos2* nitric oxide synthase 2, *Tnf-α* tumor necrosis factor-α
